# Phenethyl isothiocyanate and dasatinib combination synergistically reduces hepatocellular carcinoma growth via cell cycle arrest and oxeiptosis

**DOI:** 10.3389/fphar.2023.1264032

**Published:** 2023-10-04

**Authors:** Gabriele Strusi, Caterina M. Suelzu, Nicole Horwood, Andrea E. Münsterberg, Yongping Bao

**Affiliations:** ^1^ Norwich Medical School, University of East Anglia, Norwich Research Park, Norwich, United Kingdom; ^2^ School of Biological Sciences, University of East Anglia, Norwich, United Kingdom

**Keywords:** cancer therapeutics, oncology, drug development, oxeiptosis, combination therapy, dasatinib, PEITC

## Abstract

**Introduction:** Hepatocellular carcinoma (HCC) is the most common type of liver cancer, which is among the most lethal tumours. Combination therapy exploits multiple drugs to target key pathways synergistically to reduce tumour growth. Isothiocyanates have been shown to possess anticancer potential and to complement the anticancer activity of other compounds. This study aimed to investigate the potential of phenethyl isothiocyanate (PEITC) to synergise with dasatinib, improving its anticancer potential in HCC.

**Methods:** MTT, 3D spheroids and clonogenic assays were used to assess the combination anti-tumour effect *in vitro*, whereas a murine syngeneic model was employed to evaluate the combination efficacy *in vivo*. DCFDA staining was employed to evaluate the production of reactive oxygen species (ROS), while flow cytometry and Western blot assays were used to elucidate the molecular mechanism of the synergistic activiy.

**Results:** PEITC and dasatinib combination exhibited a synergistic effect *in vitro* and *in vivo*. The combination induced DNA damage and oxidative stress through the production of ROS, which led to the formation of a premature CDK1/Cyclin B1 complex associated with induction of mitotic catastrophe. Furthermore, ROS activated oxeiptosis, a caspase-independent form of programmed cell death.

**Conclusion:** PEITC showed to enhance dasatinib action in treating HCC with increased production of ROS that induced cell cycle arrest followed by mitotic catastrophe, and to induce oxeiptosis. These results highlight the role that ITCs may have in cancer therapy as a complement of clinically approved chemotherapeutic drugs.

## Graphical Abstract

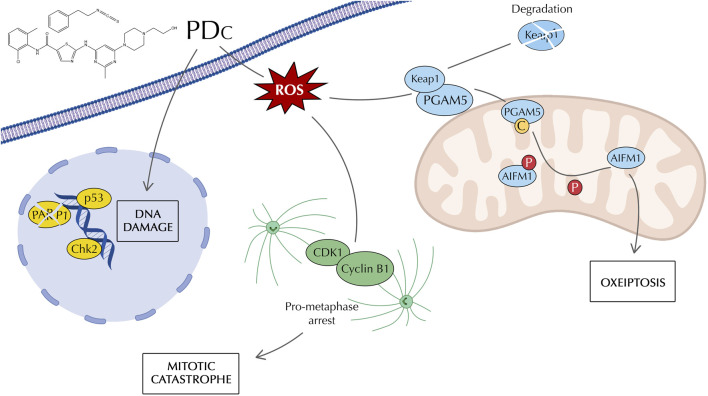

## 1 Introduction

Liver cancer is the second most lethal tumour, with a survival rate of 13% (El-Serag, 2020; McGlynn et al., 2021). Every year, more than 6,000 people are diagnosed with liver cancer (Cancer Research UK). The mortality rate has increased by 40% in the last decade, and the prediction is that by 2040, there will be 1.3 million deaths related to liver cancer (Zhang et al., 2022a). Hepatocellular carcinoma (HCC) is the most common type of liver cancer, and the first-choice modality treatments at early stages are tumour resection and liver transplant. However, the diagnosis usually arrives after the onset of severe symptoms, indicating an advanced stage of the disease. The first drug approved for the treatment of HCC is sorafenib, which has been the only treatment for more than a decade. Even though there has been progress over the last 5 years, the efficacy and the number of available HCC treatments are not comparable to the one of approved drugs for the treatment of other types of cancer (Zhang et al., 2022a; Scott et al., 2023). Therefore, there is an urgent need to develop new therapies for HCC.

Isothiocyanates (ITCs) are compounds derived from the hydrolysis of glucosinolates found abundant in cruciferous vegetables of the Brassicaceae family (Vaughn and Berhow, 2005). An inverse association between the consumption of cruciferous vegetables and the risk of cancer onset has been observed, suggesting ITCs should be explored in cancer research (Verhoeven et al., 1996). Numerous studies showed that ITCs also play a role in reducing cancer growth (Lenzi et al., 2014). In particular, ITCs activate different pathways that reduce HCC growth and its metastatic potential (Zhang et al., 2022b). Moreover, ITCs improved the anticancer activity of several chemotherapeutic agents, allowing their repurposed use in non-small cell lung cancer (Zhang et al., 2020), breast cancer (Kaczyńska and Herman-Antosiewicz, 2017), and gastric cancer (Yi et al., 2021).

Dasatinib, a potent multi-targeted inhibitor approved for the treatment of chronic myeloid leukaemia, is in trials for treatment of different types of solid tumour (Araujo and Logothetis, 2010). Dasatinib affected cell proliferation in different HCC cell lines by targeting Src/FAK and Akt pathways (Chang and Wang, 2013a; Chang and Wang, 2013b). On the other hand, a phase II trial of dasatinib treatment of un-resectable HCC was terminated because of discouraging results (Dasatinib, 2015). Further studies showed that the effect of dasatinib in HCC *in vitro* and *in vivo* is limited by the over-activation of FAK pathway and that its anticancer potential can be enhanced in combination with FAK inhibitors (Liu et al., 2018). Indeed, the combination of dasatinib and rosuvastatin showed a synergistic effect with the reduction of HCC *in vivo* (El Sayed et al., 2018). Therefore, dasatinib treatment of HCC can be improved when combined with other agents (Finn et al., 2013) with particular potential in sorafenib-resistant tumours (Chang and Wang, 2013b).

Cancer therapy research has now shifted towards the strategy of combination therapy, and in particular, recent clinical trials showed that the association of multiple drugs appear to be the most promising approach for the treatment of HCC (Zhang et al., 2022a). Indeed, a new first-line treatment for HCC has been recently approved, combining Atezolizumab and Bevacizumab for an increased potency, compared to the two monotherapies (Finn et al., 2020). Preliminary screening between several isothiocyanates and dasatinib showed that the addition of phenetyl isothiocyanate to dasatinib leads to a synergistic effect with increased cytotoxicity towards HCC cells. The combination of dasatinib with ITCs has never been explored for the treatment of solid tumours. Here, we investigate whether the broad ITCs anticancer activity can be exploited to improve the action of dasatinib to be repurposed for the treatment of HCC. Preliminary experiments showed that combination of phenethyl isothiocyanate (PEITC) and dasatinib, termed PDc, exhibits cytotoxicity towards HepG2 cells. Therefore, this study used pre-clinical *in vitro* and *in vivo* models to characterise the effects of PDc. Furthermore, we elucidate the mechanism of action and demonstrate the synergistic anticancer activity of the drug combination.

## 2 Materials and methods

Details of suppliers and catalogue numbers are provided in the Supplementary Table S1.

### 2.1 Cell lines

HepG2 and Hepa 1-6 cells were cultured in DMEM supplemented with 10% FBS, 1% L-glutamine (200 mM), penicillin (100 U/ml) and streptomycin (100 μg/mL), at 37°C, 5% (v/v) CO_2_.

### 2.2 Cell viability assay

Cells (HepG2 7 × 10^3^ cells/well, Hepa1-6 5 × 10^3^ cells/well) were seeded in 96-well plates and received the drug treatment once 80% confluent. After 24 h, 100 µL of 10% 5 mg/mL MTT in DMEM was added to each well and incubated at 37 °C for 1 h. Formazan crystals were then dissolved in 100 µL of DMSO per well. The absorbance was determined using a FLUOstar omega plate reader at 550 nm. Combination index (CI) values was calculated using 1 and Compusyn software.

### 2.3 Colony formation assay

Cells (HepG2 4 × 10^5^ cells/well) were seeded in 6-well plates. After 24 h, drug treatment was applied. After 24 h the medium was replaced with fresh DMEM. Cell cultures were maintained for 18 days with medium changed every 5 days. The colonies formed were fixed with 10% buffered formalin and stained with 0.1% crystal violet. Macroscopic images were acquired using a document scanner (IM IC5500). The colony area was calculated using ImageJ 1.53k software with the macro “Colony area” described in Guzmán *et al.* work (Guzmán et al., 2014).

### 2.4 3D spheroids model

Cells (HepG2 1.25 × 10^3^ cells/well) were seeded in CELLSTAR^®^ cell repellent U bottom 96-well plates with addition of ECM Gel 0.1 mg/mL. The plates were centrifuged for 5 min at 210 x g at room temperature. After 3 days, images were taken with EVOS M5000 Imaging System and drug treatments were applied. On day six, images were taken for growth quantification with ImageJ and spheroids were stained with Calcein AM (1 µM) and Ethidium homodimer-1 (3 µM), for viability evaluation. Images were acquired using a Leica DMI3000 B microscope.

### 2.5 DNA damage

Cells (HepG2 1.5 × 10^5^ cells/well) were seeded on glass coverslips and placed inside 6-well plates; treatments were applied once cells reached 80% confluence. After 24 h, wells were fixed with 10% buffered formalin and 0.1% Triton X-100. Coverslips were mounted with 25 µL of ProLong™ Gold Antifade Mountant with DAPI and morphological changes were observed using a Leica DMI3000 B fluorescence microscope.

### 2.6 Cell cycle analysis

Cells (HepG2 3.2 × 10^5^ cells/well) were seeded in 6-well plates and treated once 80% confluent. After 24 h, cells were detached, washed, and fixed with cold 70% ethanol. Fixed cells were washed and stained with 500 µL of BD Pharmingen™ PI/RNase staining buffer. Samples were analysed with a BD FACSymphony A1™ flow cytometer and 10,000 events were collected. Data were analysed using ModFit LT 6.0.11 software. Gating strategy is presented in Supplementary Figure S1.

### 2.7 Apoptosis evaluation

Cells (HepG2 1.5 × 10^5^ cells/well, Hepa 1–6 9 × 10^5^ cells/well) were seeded in 12-well plates and treated once 80% confluent. After 24 h, cells were detached and stained with the Invitrogen Annexin V/PI kit as per manufacturer instructions. Samples were analysed with a Cube 6 flow cytometer and 10,000 events were collected. Data were analysed using FlowJo 10.4 software. Compensation controls and positive controls are presented in Supplementary Figure S2.

### 2.8 ROS quantification

Cells (HepG2 2.5 × 10^4^ cells/well) were seeded in a 96-well plate. After 24 h, cells were stained with abcam DCFDA - Cellular ROS Assay Kit according to the manufacturer instructions. Tert-Butyl hydroperoxide (TBHP) was used as positive control. Cells were treated for 4 h. The plate was incubated in a FLUOstar omega plate reader at 37°C, 5% CO_2_ for 4 h, with readings (Ex/Em = 485/535) obtained every 30 min. Fluorescent intensity values were normalised to time 0 (T0) readings. Representative images were acquired after 4 h of incubation with the Leica DMI3000 B microscope.

### 2.9 Western blotting

Cells were treated for 24 h, if not differently specified and total protein content was extracted from treated cells using a solution of 20 mM Tris-HCl (pH 8), 150 mM NaCl, 2 mM EDTA, 10% glycerol, 1% Nonidet P40 added with Cell signaling Protease Inhibitor Cocktail (100X) and Pierce™ Phosphatase Inhibitor Mini Tablets. Protein quantification was performed using Bradford reagent. The protein concentration was assessed with a FLUOstar omega plate reader at 595 nm. Between 20–40 µg of sample was loaded onto SDS-polyacrylamide gels and separated in a Mini Protean Tank filled with TruPAGE™ TEA-Tricine SDS Running Buffer. Molecular weight was determined by PageRuler™ Plus. Separated proteins were transferred into a PVDF membrane using a semi dry Trans-Blot^®^ Turbo™ Transfer System. The membrane was blocked with Intercept TBS blocking buffer and incubated with primary antibody solution overnight at 4 °C. After washing, the membrane was incubated with a secondary antibody IRDye^®^ solution for 1 h on low rocker speed at room temperature. Finally, the membrane was washed and scanned with an Odyssey CLx Imaging System. The protein band density was quantified using Image Studio Lite 5.2.5 software and normalised to the signal of β-actin. Details of antibodies used are provided in Supplementary Table S2.

### 2.10 Immunocytochemistry of ⍺-tubulin

HepG2 cells were seeded on 15 mm coverslips in 24 well plates (5 × 10^4^ cells/well). After 24 h cells were treated for further 24 h, fixed with 10% formalin for 15 min and permeabilised with 0.1% Triton X-100. Blocking buffer (5% goat serum PBS) was added for 2 h on a rocker at room temperature. ⍺-Tubulin antibody solution was incubated overnight on a rocker at 4°C. The wells were rinsed in washing buffer (0.1% Tween 20 PBS) and then washed twice for 10 min on a rocker at room temperature. Cells were incubated with secondary antibody solution for 1 h on a rocker at room temperature. The wells were rinsed in washing buffer and then washed twice for 5 min on a rocker at room temperature. Coverslips were mounted with 10 µL of ProLong™ Gold Antifade Mountant with DAPI. Microtubule morphology was observed using a Leica DMI3000 B microscope. Details of antibodies used are provided in Supplementary Table S3.

### 2.11 Subcutaneous syngeneic HCC murine model

Hepa 1-6 cells (5 × 10^6^ cells in 100 µL MEM) were injected subcutaneously in the right flank of 40 9–10-week-old female C57BL6 mice purchased from Envigo ltd. (UK). After 10 days, mice were randomised into 4 groups with 10 mice per group once the tumour volume reached 170 mm^3^. Individual animals were administered with 100 µL of corn oil (5 days/week) by oral gavage. The treatment groups were as follows: vehicle 2% DMSO; 40 mg/kg PEITC +2% DMSO; 10 mg/kg dasatinib in 2% DMSO; or 40 mg/kg PEITC +10 mg/kg dasatinib. Animals were marked with different colours depending on treatment group and housed in mixed cages to exclude any environmental influence. Animal weight and tumour size were measured twice/week. Tumour volume was calculated using the following formula: volume = 0.52 x (L x w^2^). After 3 weeks of treatment, the mice were euthanised and tumours were excised and measured. The experiment was run by two unblinded investigators. Animals that did not develop a tumour were excluded from the results. No statistical analysis was used for *a priori* sample size calculation. All animal work was carried out in accordance with regulations established by the UK Home Office (London, United Kingdom) and the Animal Scientific Procedures Act of 1986 under the Home Office Project Licence PP5500397.

### 2.12 Statistical analysis

Data presented are expressed as mean ± SEM. Statistical analysis was performed using Prism 9 software. Normal distributed data sets were subjected to one-way ANOVA with Tukey’s *post hoc* test for multiple comparison between three or more groups, or to the student’s t-test for two groups comparison. Non-normal distributed datasets were subjected to the non-parametric test Kruscal-Wallis with Dunn’s multiple comparison between three or more groups, or to the Mann-Whitney U test for two groups comparison. Statistical difference in *in vivo* tumour growth rate was defined by the comparison of the slope of the linear regression described by tumour volume data points over time. Sample size (n) represents the number of independent experiments or biological replicates for the *in vivo* study. The degrees of significance are indicated as *p ≤ 0.05, **p ≤ 0.01, ***p ≤ 0.001, ****p ≤ 0.0001.

## 3 Results

### 3.1 PDc inhibits HepG2 growth and proliferation in a synergistic manner

**FIGURE 1:**
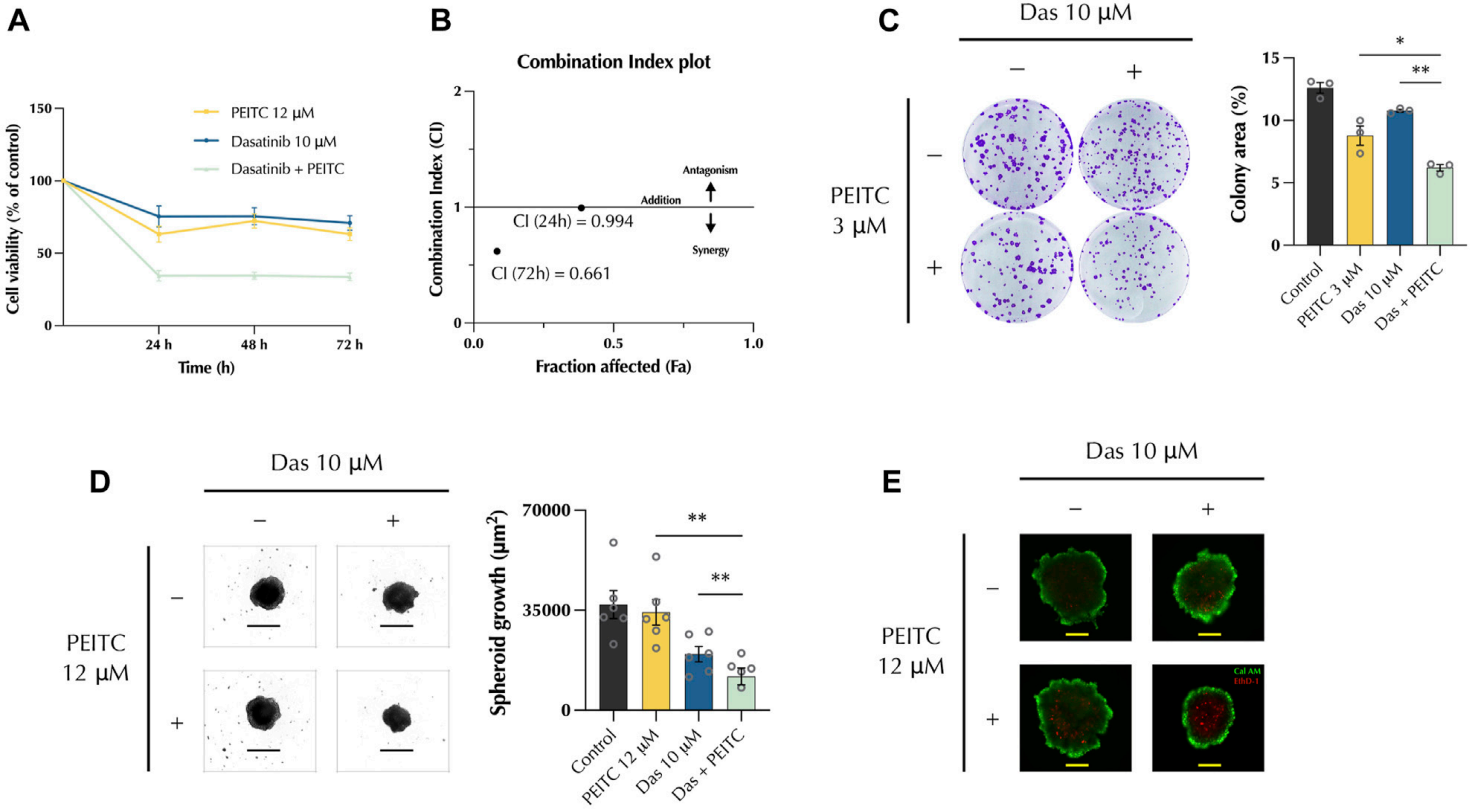
PDc synergistically inhibits HCC cells HepG2 growth and proliferation **(A)** HepG2 cell viability (mean ± SEM, n = 3) assessed by MTT proliferation assay. **(B)** Combination Index value at 24 h and 72 h calculated with CompuSyn software. **(C)** Representative images of colony formation assays and their quantification (mean ± SEM, n = 3); the degrees of significance are indicated as *p < 0.05, **p < 0.01, ****p < 0.0001 and calculated with one-way ANOVA and Tukey’s *post hoc* multiple comparisons test. **(D)** 3D spheroids brightfield (4x, scale bar 500 µm) and area quantification (mean ± SEM, n = 4). The degrees of significance are indicated as *p < 0.05, **p < 0.01 calculated with non-parametric Kruskal-Wallis test with Dunn’s multiple comparisons test. **(E)** 3D spheroids cell viability staining; dead cells and viable cells are stained with EthD-1 (red) and Calcein AM (Green), respectively (10x, scale bar 200 µm).

HepG2 cells were treated with either PEITC (12 μM), Dasatinib (10 μM), or their combination and their combinatorial effects were tested. Cell viability was measured after 24, 48, and 72 h with MTT assay. This showed a significant decrease of cell viability in the combination treatment (Figure 1A). To determine the nature of the interaction between the two compounds, the Chou-Thalalay method was employed to calculate the combination index. Values below 1.0 indicates that the drugs work in synergy, whereas values above 1.0 indicates an antagonist effect. The combination index plot (Figure 1B) showed a value of 0.994 at 24 h and 0.661, indicating that the drugs synergise. A colony formation assay determined the ability of PDc to inhibit HepG2 cell proliferation. PDc treatment reduced the ability of HepG2 to form new colonies from single cells, and colony area was smaller (Figure 1C). Next, we used HepG2 3D spheroids to verify if the drug combination could effectively penetrate a tumour mass. Spheroids were formed by centrifugation and after 3 days drug treatments were applied, and spheroids were measured to assess their growth. PDc treatment significantly reduced spheroid growth (Figure 1D). A dead/live staining tested effects on cell viability and PDc treated spheroids exhibited an increased number of dead cells compared to the single drug treatments, indicating increased toxicity of the drug combination towards hepatoma HepG2 cells (Figure 1D).

### 3.2 PDc induces DNA damage response through p53 expression and promotes apoptosis

**FIGURE 2:**
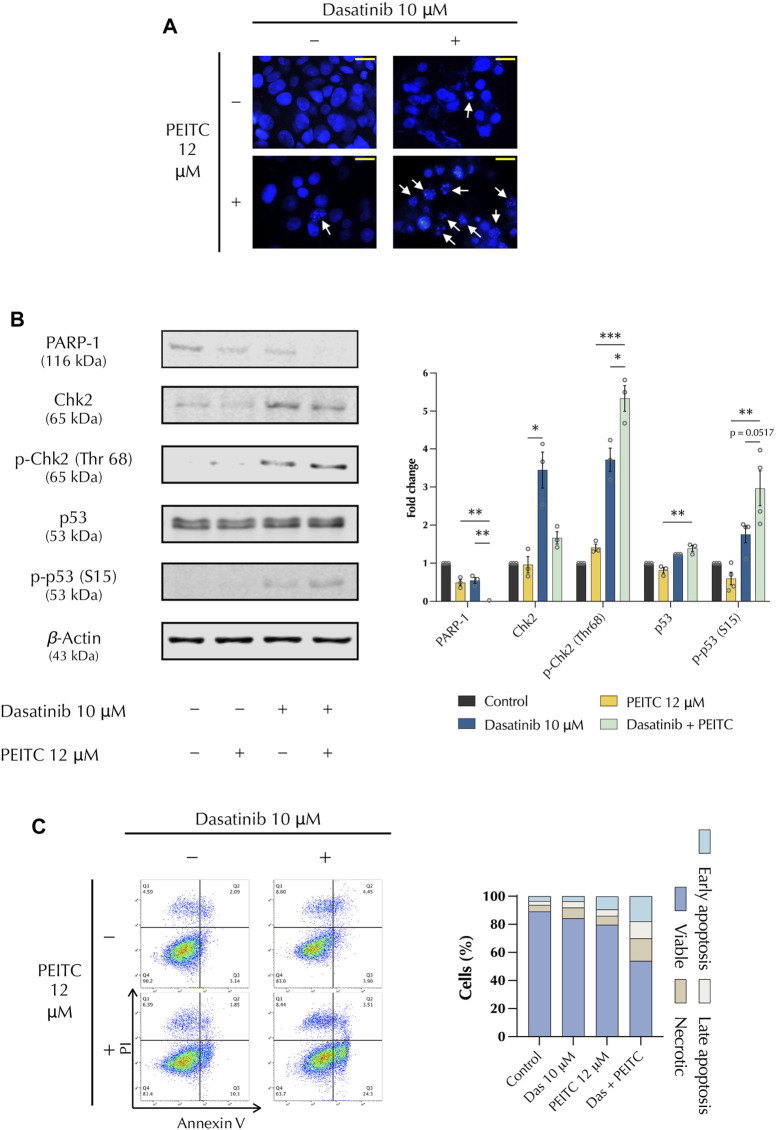
PDc induces DNA damage response through the expression of tumour suppressor p53 and promotes apoptosis. **(A)** Representative images of DAPI-stained cells; arrows indicate cell blebbing and chromatin condensation (100x, scale bar 20 µm). **(B)** Western blot representative images of PARP1, Chk2, and p53 analysis; fold change of proteins normalised to β-Actin expression (mean ± SEM, n = 3). The degrees of significance are indicated as *p < 0.05, **p < 0.01, ***p < 0.001 and calculated with one-way ANOVA and Tukey’s *post hoc* multiple comparison test. **(C)** Apoptosis assay FlowJo representative images from Annexin V and PI staining.; representative results from 3 independent experiments.

The induction of DNA damage and inhibition of DNA repair mechanisms can be exploited to target cancerous cells. Therefore, the effect of PDc on DNA damage was investigated compared to the single drug treatments after 24 h of treatment (Figure 2). DAPI staining showed chromatin condensation and the presence of apoptotic bodies, thus confirming induction of DNA damage (Figure 2A). A fundamental marker in the DNA damage response is the p53 tumour suppressor (Finn et al., 2020), which regulates numerous transcription factors and is involved in DNA repair, cell cycle regulation, and apoptosis induction. p53 is activated by several factors, such as the checkpoint kinase 2 (Chk2), which is in turn activated by double strand breaks (DSB). Another factor that plays a role in DNA damage is PARP. The inhibition of PARP has a fundamental role in cancer therapy as inhibit the ability of cancerous cells to repair DSBs. Western blot analysis showed that PDc led to the reduction of total PARP-1 accumulation, increased the phosphorylation of Chk2 (Thr 68), and the activation of p53 (Ser 15) (Figure 2C). To determine whether DNA damage was accompanied by apoptosis induction, a staining of Annexin V and propidium iodide was performed. Flow cytometry analysis revealed an increase of both early and late apoptosis in PDc-treated cells (Figure 2D).

### 3.3 PDc reduces HepG2 proliferation through ROS induced apoptosis

**FIGURE 3:**
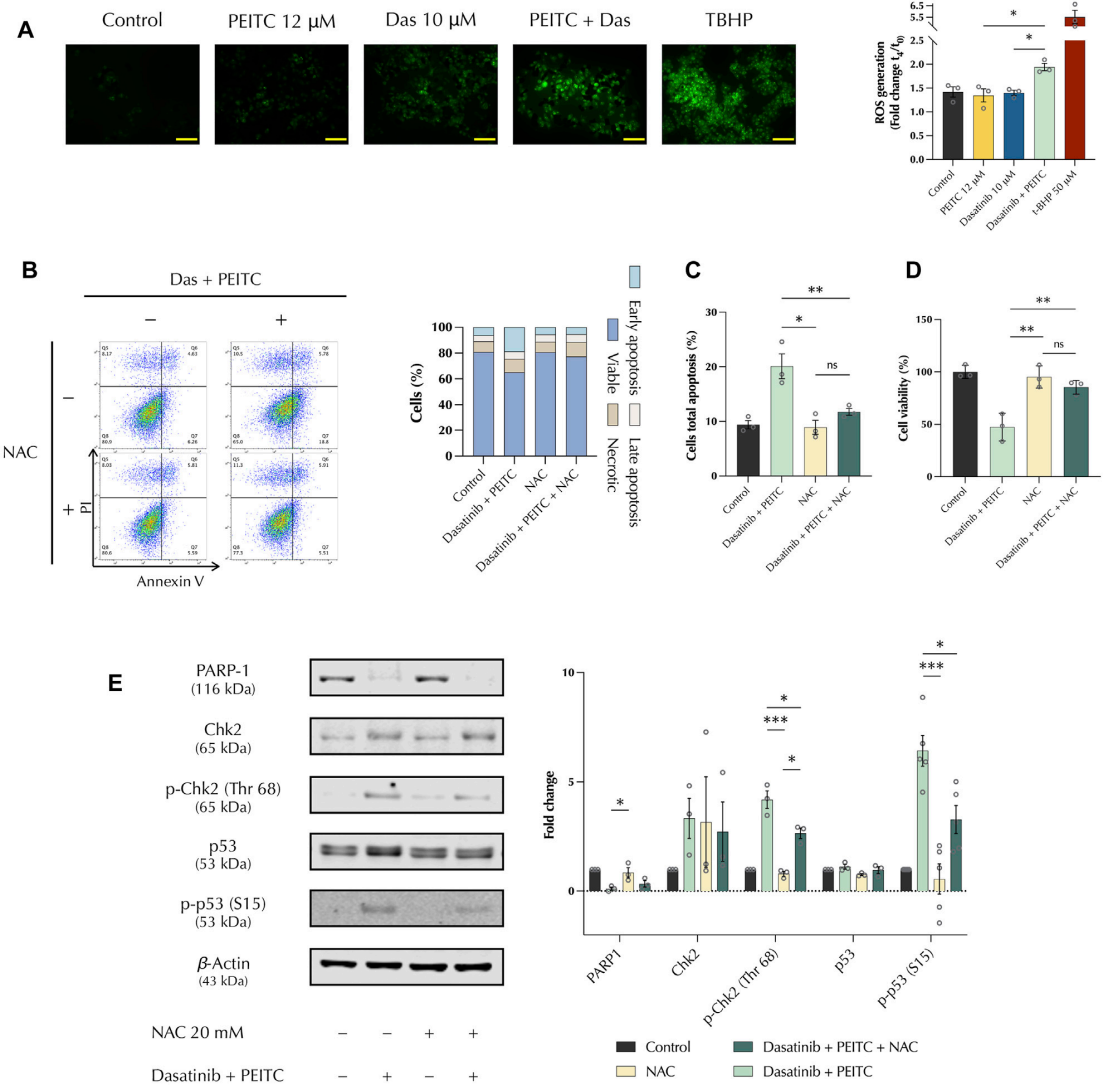
PDc reduces HepG2 proliferation through ROS-induced apoptosis **(A)** Left, representative images of DCFDA staining after 1 h of treatment (scale bar 100 µm); right, ROS quantification after 6 h of treatment shown as fold change (mean ± SEM, n = 3). The degrees of significance are indicated as **p < 0.01, ***p < 0.001, calculated with one-way ANOVA and Tukey’s *post hoc* multiple comparison test. **(B)** Left, apoptosis assay FlowJo representative images for Annexin V and PI staining; right, representative results, n = 3. **(C)** Cells’ total apoptosis assessed after pre-treatment with 20 mM NAC (mean ± SEM, n = 3). The degrees of significance are indicated as *p < 0.05, **p < 0.01 and calculated with one-way ANOVA and Tukey’s *post hoc* multiple comparison test. **(D)** Cells’ viability determined after pre-treatment with 20 mM NAC (mean ± SEM, n = 3). The degrees of significance are indicated as *p < 0.05, **p < 0.01, calculated with one-way ANOVA and Tukey’s *post hoc* multiple comparison test. **(E)** Western blot representative images of PARP1, Chk2, and p53 analysis after pre-treatment with 20 mM NAC; fold change of proteins normalised to β-Actin expression (mean ± SEM, n = 3). The degrees of significance are indicated as *p < 0.05, **p < 0.01, ***p < 0.001 and calculated with one-way ANOVA and Tukey’s *post hoc* multiple comparison test.

Oxidative stress plays a fundamental role in chemotherapy as cancerous cells present a redox imbalance that make them susceptible to the increased induction of reactive oxygen species (ROS) (Guzmán et al., 2014). The production of ROS *in vitro* was investigated. DCFDA staining showed that PDc increased the production of H2O2 compared to the single treatments. This was confirmed by immunofluorescence, which showed an increased signal from PDc-treated cells (Figure 3A). Pre-treatment of 2 h with the ROS scavenger, N-acetyl-cystein (NAC), confirmed that ROS are involved in PDc anticancer activity. NAC pre-treatment abolished apoptosis induction (Figures 3B,C) and recovered HepG2 cell viability after PDc treatment (Figure 3D). NAC pre-treatment significantly reduced the activation of both p53 and Chk2 proteins, but it did not affect the degradation of PARP1 previously observed (Figure 3E).

### 3.4 PDc induces G2/M phase cell cycle arrest through CDK1/Cyclin B1 complex

**FIGURE 4:**
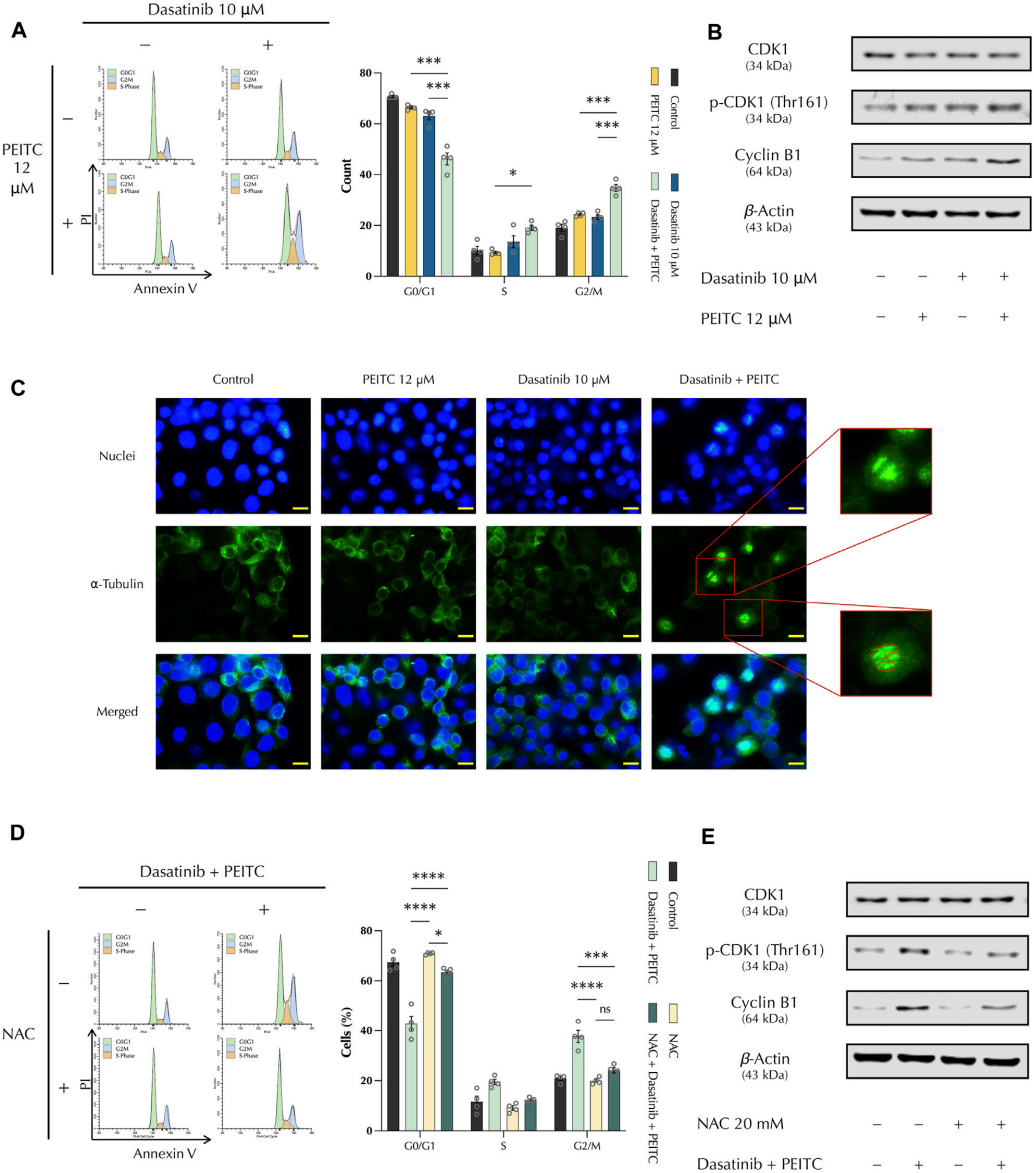
PDc induces G2/M phase cell cycle arrest through CDK1/Cyclin B1 complex **(A)** Left, representative images of cell cycle assay from ModFit LT PI staining; right, cell cycle phases quantification (mean ± SEM, n = 4). The degrees of significance are indicated as *p < 0.05, ***p < 0.001, calculated with one-way ANOVA and Tukey’s *post hoc* multiple comparison test. **(B)** Western blot analysis of CDK1 and cyclin B1; the results presented are representative of three independent protein extractions. **(C)** Representative images of α-tubulin-stained cells treated with PDc and single drugs (100x, scale bar 20 µm). The red circles indicate the three different poles from which the mitotic spindle fibres ramify, indicating an abnormal mitotic spindle formation. **(D)** Left, cell cycle assay representative images from ModFit LT PI staining after pre-treatment with N-acetylcysteine; right, cell cycle phases quantification (mean ± SEM, n = 4). The degrees of significance are indicated as *p < 0.05, ***p < 0.001, ****p < 0.0001, calculated with one-way ANOVA and Tukey’s *post hoc* multiple comparison test. **(E)** Western blot analysis of CDK1 and cyclin B1 after pre-treatment with N-acetylcysteine; the results presented are representative of three independent protein extractions.

DNA damage and apoptosis induction are related to cell cycle regulation. Hence, cell cycle phases were analysed to assess if the drug combination induced a cell cycle arrest, controlling the proliferation of HepG2 cells. Propidium iodide staining was used with flow cytometry to quantify the number of cells present on each phase of the cell cycle. This showed that PDc led to a significant increase of the number of tetraploid cells, indicating a cell cycle arrest at the G2/M phase checkpoint (Figure 4A). To investigate the mechanism of action, Western blot was employed to study the major factors involved in G2/M phase arrest. Protein expression analysis showed that PDc induced the accumulation of Cyclin B1 and over-activation of cyclin-dependent kinase 1 (CDK1) (Figure 4B). It has been shown that over-activation of CDK1 and accumulation of Cyclin B1 lead to the formation of a complex responsible for premature entry into mitosis (Castedo et al., 2002; Choi and Zhu, 2012), which induces cell cycle arrest and mitotic catastrophe (Vakifahmetoglu et al., 2008). Immunostaining of ⍺-tubulin showed that PDc-treatment resulted in chromatin condensations surrounded by aberrant mitotic spindle fibres that ramify from three mitotic poles, which indicate an abnormal spindle formation typically observed in mitotic catastrophe (Figure 4C). Next, the cell cycle analysis was repeated with NAC pre-treatment to examine whether ROS production played a role in cell cycle arrest. The results underlined that the ROS scavenger protected HepG2 cells from the PDc mediated induction of cell cycle arrest (Figure 4D). In addition, protein expression analysis confirmed the role of ROS induction in PDc mediated cell cycle arrest, with reduced accumulation of Cyclin B1 and a significant reduction of CDK1 activation (Figure 4E).

### 3.5 PDc induces oxeiptosis in HepG2 hepatoma cells

**FIGURE 5:**
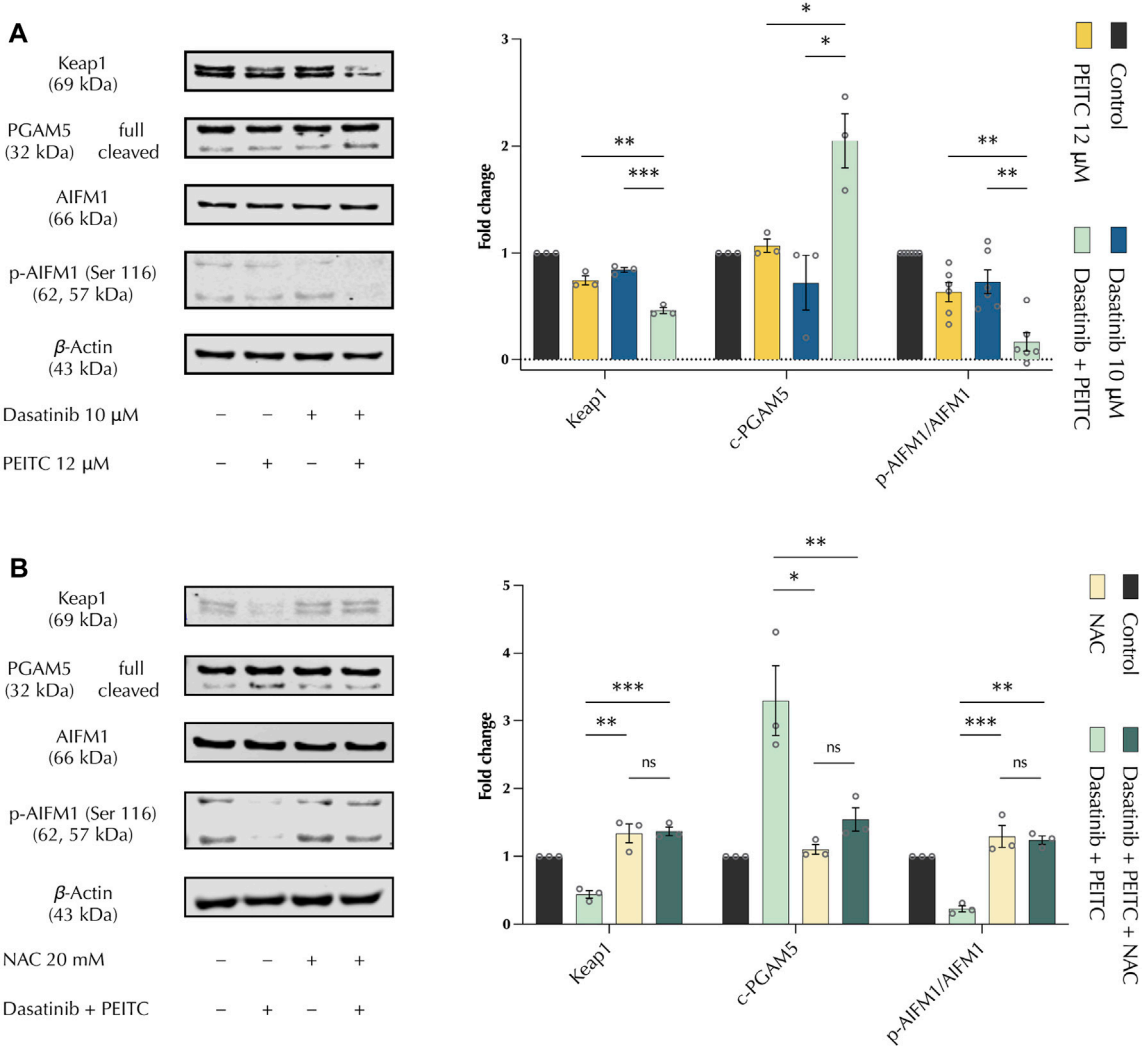
PDc induces oxeiptosis in HepG2 HCC cells. **(A)** Western blot analysis of oxeiptosis markers Keap1, PGAM5, AIFM1; the results presented are representative of at least three independent protein extractions (left). Fold change to control normalised to β-Actin (mean ± SEM, n ≥ 3) (right); the degrees of significance are indicated as *p < 0.05, **p < 0.01, ***p < 0.001, calculated with one-way ANOVA and Tukey’s *post hoc* multiple comparison test. **(B)** Western blot analysis of oxeiptosis markers Keap1, PGAM5, AIFM1 after NAC pre-treatment; the results presented are representative of three independent protein extractions (left). Fold change to control normalised to β-Actin (mean ± SEM, n = 3) (right); the degrees of significance are indicated as *p < 0.05, **p < 0.01, ***p < 0.001 calculated with one-way ANOVA and Tukey’s *post hoc* multiple comparison test.

To determine the mechanism of apoptosis induction, the expression of apoptosis markers was evaluated using the Human Apoptosis Proteome Profiler Antibody Array. The protein array did not show any significant change between single drug and PDc treatments that could explain the increased apoptotic induction previously observed with the apoptosis detection assay (Supplementary Figure S3A). Next, to confirm the results of the antibody array, Western blot was employed to evaluate the expression of Caspase −3, −9 and cytochrome c. The protein expression analysis confirmed the absence of Caspase activation and suggested a slight increase of cytochrome c accumulation, which resulted non-significant in the comparison between single drug and combination treatments (Supplementary Figure S3B). Since ROS is involved in PDc-induced DNA damage and cell cycle arrest, we evaluated the expression of oxeiptosis markers. Oxeiptosis is a non-canonical, caspase-independent form of apoptosis characterised by the presence of high levels of ROS that activate the cell death mechanism (Vakifahmetoglu et al., 2008). Western blot analysis showed that PDc affects the expression of all identified markers of oxeiptosis. PDc decreased accumulation of Keap1, increased both the cleavage of PGAM5 and the dephosphorylation of AIFM1 (Figure 5A). Moreover, NAC pre-treatment showed that PDc mediated ROS production is responsible for the effects on oxeiptosis markers. Thus, ROS associated oxeiptosis is at least in part responsible for the anticancer activity in hepatoma HepG2 cells (Figure 5B).

### 3.6 PDc suppresses tumour growth *in vivo* in a synergistic manner

**FIGURE 6:**
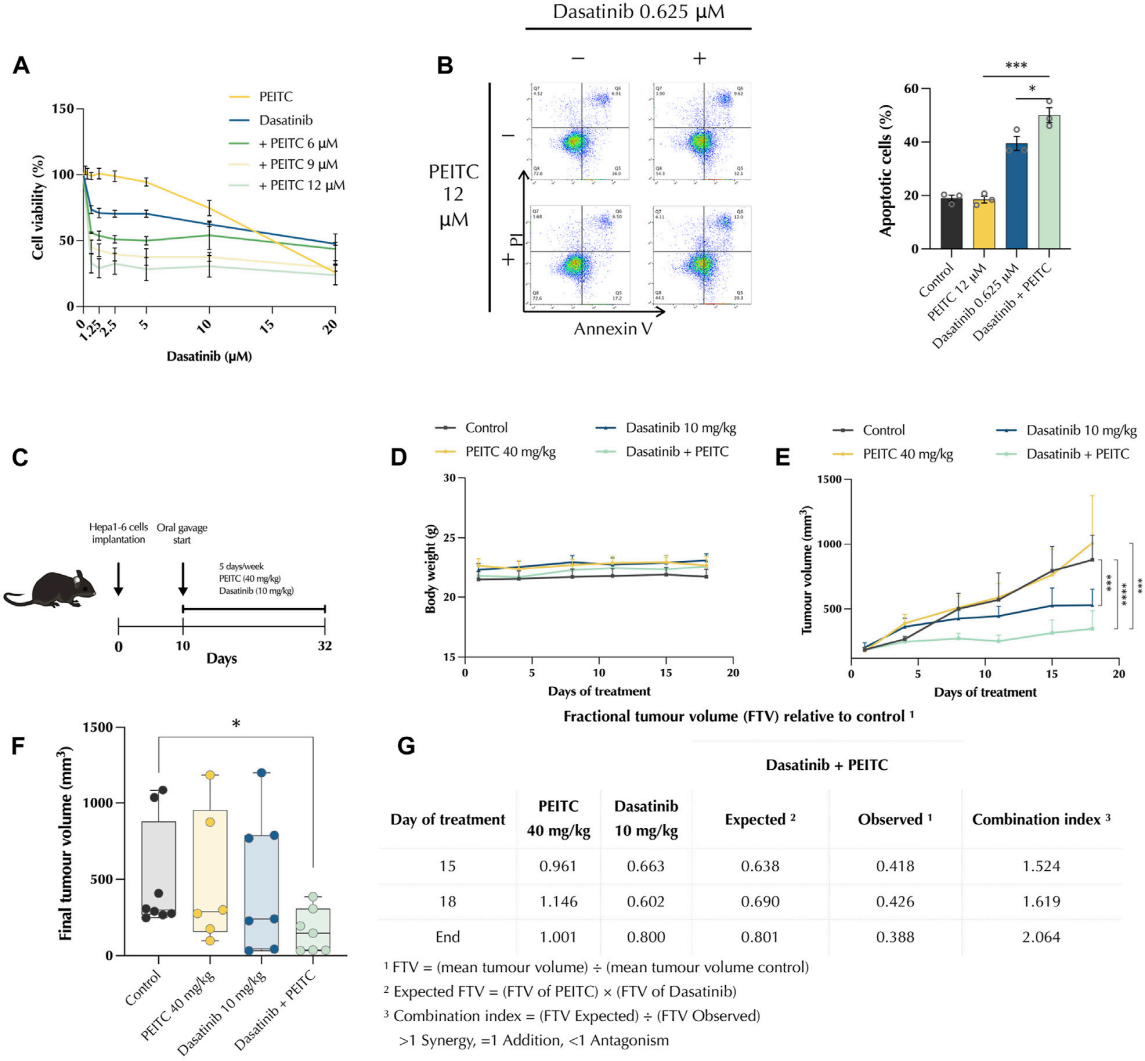
PDc reduces tumour growth *in vivo* in a murine model. *In vitro* effect Effect of PDc on Hepa 1-6 cells *in vitro* **(A)** proliferation, **(B)** apoptosis induction. **(C)** Diagram of *in vivo* experimental conditions treatment regime. **(D)** Mice body weight over the treatment time. **(E)** Tumour volume over the treatment time (mean ± SEM, control n = 9, PEITC n = 6, dasatinib n = 8, P + D n = 8). The degrees of significance are indicated as ***p < 0.001, ****p < 0.0001, calculated with linear regression. **(F)** Final tumour volume normalised to starting volume. The degree of significance is indicated as *p < 0.05 and calculated with non-parametric Kruskal-Wallis test with uncorrected Dunn’s test. **(G)** Drug combination synergistic effect was calculated with combination index relative to fractional tumour volume.

To evaluate whether PEITC can improve the tumour growth inhibition of dasatinib in HCC, a mouse syngeneic subcutaneous model was employed. First, we tested the effects of PDc on the murine Hepa 1-6 cell line *in vitro*; MTT assay and Annexin V/propidium iodide assay were employed to assess effects on cell viability and apoptosis induction respectively. PDc reduced Hepa 1-6 cell viability in a synergistic manner (Figure 6A), as confirmed by CI values (Supplementary Table S4). A concentration of 12 uM PEITC, together with only 0.625 μM dasatinib led to maximum reduction of Hepa 1-6 cell viability. Moreover, apoptosis assay revealed a significant increase in the number of apoptotic cells in PDc treatment (Figure 6B). Subsequently, C57BL6 mice were subcutaneously injected with 5 × 106 Hepa 1-6 cells; after tumour formation mice were treated for 3 weeks and then sacrificed to collect the tumours (Figure 6C). The treatments did not show any detrimental effects on body weight (Figure 6D), which remained stable throughout the experiment. However, PDc reduced tumour growth compared to vehicle control group and single drug treated-groups (Figure 6E). The PDc treated excised tumours showed a significant difference in terms of volume compared to vehicle control group (Figure 6F). Calculation of fractional tumour volume highlighted that the drug combination elicited a combination index of 2.064, indicating strong synergism (Figure 6G).

## 4 Discussion

In this study, the mechanism of action and the preclinical efficacy of the synergistic interaction between the anticancer drug dasatinib and PEITC has been described. Treatment with PDc inhibited the growth of HCC HepG2 cells, with increased production of ROS. The oxidative imbalance was demonstrated to be responsible for the induction of DNA damage, cell cycle arrest and oxeiptosis. Furthermore, PDc showed promising results *in vivo* with the reduction of hepatoma growth in a murine syngeneic model. The results provide insights into the role of PEITC in improving the anticancer activity of approved anticancer agents for the treatment of HCC.

The investigation of ROS production *in vitro* using DCFDA staining highlighted that PDc led to an increased ROS production and the pre-treatment with the antioxidant molecule NAC proved that ROS have a fundamental role in PDc increased anticancer activity. PEITC is known for its ability to induce oxidative imbalance through depletion of GSH (Hong et al., 2015), whilst dasatinib relies on oxidative stress for improved anticancer activity when combined with other drugs (Wang et al., 2022). PDc was shown to lead to an imbalance in the GSSG/GSH ratio in the first 6 h of treatment (Supplementary Figure S4), which could explain the increased production of ROS compared to single drugs treatment. It is widely known that ROS production can be exploited to trigger cell cycle arrest and programmed cell death to reduce tumour growth due to the abnormal redox status of cancerous cells (Perillo et al., 2020).

As well as inhibiting the growth of HepG2 cells in 2D and 3D and reducing the cell clonogenic potential, PDc was shown to induce chromating condensation and cell blebbing and activation of DNA damage response through upregulation of the oncosuppressors p53 and Chk2. It has been shown that Chk2 activation is associated with cellular response to DNA damage in the ATM-Chk2-p53 pathway (Hirao et al., 2000). ATM phosphorylates both p53 Ser-15 and Chk2 Thr-68 playing a role in cycle arrest and apoptosis (Cao et al., 2006). P53 activation is known to play a role in the induction of cell cycle arrest at the G1 phase as it regulates the expression of. PDc showed to activate Chk2 and p53 but with no induction of G1 arrest. Indeed, Lee *et al.* showed that Chk2 can activate p53, independently of Atm- and p53-dependent G_1_ arrest, to induce DNA damage cell death (Jack et al., 2002). The increased cell blebbing and chromatin condensation, together with the activation of both p53 and Chk2, indicates an increased DNA damage response. An essential factor in DNA damage response is PARP, a member of the poly (ADP-ribose) polymerases protein family (Wang et al., 2012). PARP is activated through proteolytic cleavage to induce DNA repair and it is found highly expressed in HCC. Recently, the combination of dasatinib and PARP inhibition has been identified as a novel synthetic lethal strategy to tackle HCC growth (Sun et al., 2022). Indeed, combined treatment of olaparib and dasatinib showed an increased activation of Chk2 and inhibition of HCC growth *in vitro* and *in vivo* [31]. As PEITC has already been associated with DNA damage through p53 activation and degradation of PARP (Tang et al., 2011), the combined treatment of dasatinib and PEITC seems a valid strategy to induce DNA damage and reduce cancerous reparative processes.

Cancerous cells are known for their capacity of uncontrolled growth and ability to avoid growth suppressors (Hanahan and Weinberg Robert, 2011). PDc induced a cell cycle arrest with an increase of tetraploid cells (G_2_/M phase) and this can be associated with the increased activation of CDK1 and accumulation of Cyclin B1. CDK1 is known to interact with Cyclin B1 to form an active heterodimer that is required for the cell cycle progression to the mitosis phase (Castedo et al., 2002). However, it has been shown that a premature activation of CDK1 can lead to mitotic catastrophe after external-induced DNA damage. In fact, it has been previously outlined that 2-methoxyestradiol induced microtubule disruption, which led to the formation of CDK1/Cyclin B1 complex responsible for mitotic catastrophe and cell death (Choi and Zhu, 2012). Mitotic catastrophe is generally associated with anti-microtubular drugs and Chk2 activation after DNA damage (Castedo et al., 2002; Shang et al., 2014). PEITC is known to induce degradation of tubulin through covalent binding and ROS production (Mi et al., 2008; Yin et al., 2009). Therefore, it was hypothesised that the increased production of ROS induced by the combination of PEITC and dasatinib led to cell cycle arrest and mitotic catastrophe, as the observed effect can be reversed completely with the presence of a ROS scavenger.

Programmed cell death is a fundamental mechanism to avoid the perpetuation of anomalies that could lead to colonies of aberrant cells responsible for tumour development (Hanahan and Weinberg Robert, 2011). Therefore, cancerous cells hijack programmed cell death mechanisms to allow their uncontrolled growth. PDc increased the number of apoptotic cells in both HepG2 and Hepa 1-6 HCC cell lines. The increased apoptosis was not associated with the canonical apoptosis markers (e.g., caspases, cytochrome c), but was inhibited by NAC pre-treatment. Therefore, the attention was drawn to the novel form of apoptosis dependent on ROS production, oxeiptosis. The Nrf2-PGAM5-AIFM1 complex plays a fundamental role in oxeiptosis induction as a mechanism to cope with an increased production of ROS (Kesavardhana and Kanneganti, 2018; Scaturro and Pichlmair, 2018). This protein complex is described as a ROS sensor that mitigates damage induced by oxidative stress (Scaturro and Pichlmair, 2019). Low to mid-levels of ROS lead to the dissociation of Nrf2, which is responsible for the transcription of cytoprotective genes. However, high levels of oxidative stress induce PGAM5 release and translocation into the mitochondria, where it dephosphorylates AIFM1, the main effector of oxeiptosis. PDc was shown to decrease Keap1 accumulation, which is in contrast with what was described by Kang et al. (Kang et al., 2022), but in line with what was reported in Holze et al. and Li et al. works, in which higher levels of ROS oxidise Keap1 (Holze et al., 2018; Tsui and Li, 2023). Moreover, recent work showed that Keap1 expression increases only in the first 2 h of ROS induction and then drops drastically (Pallichankandy et al., 2023). Interestingly, PDc increased the cleavage of the phosphatase PGAM5, which was not described before in oxeiptosis induction (Holze et al., 2018; Kang et al., 2022). This could be explained by looking at the localisation of PGAM5. PGAM5 localisation is controversial as some works localise it on the outer mitochondrial membrane (Cheng et al., 2021) and others report its localisation in the internal mitochondrial membrane (Cheng et al., 2021; Wischhof et al., 2022). Increasing evidence proposes that PGAM5 can shuttle between the outer and inner membrane depending on the cell state and the mitochondrial membrane potential (Cheng et al., 2021). It has been shown that PGAM5 cleavage increases in conditions of stress, triggering different types of cell death (Cheng et al., 2021). To corroborate this thesis, Li et al. observed an increased cleavage of PGAM5 in conditions of high levels of ROS that induced oxeiptosis (Tsui and Li, 2023). Interestingly, PGAM5 cleavage happens when it is located into the internal mitochondrial membrane (Sekine et al., 2012), which is where AIFM1 is located. Finally, in line with previous works, PDc induces the dephosphorylation of AIFM1 as the main cause of oxeiptosis induction (Holze et al., 2018; Kang et al., 2022; Pallichankandy et al., 2023; Tsui and Li, 2023).

The link between mitotic catastrophe and programmed cell death remains unclear. Some researchers consider mitotic catastrophe as a type of programmed cell death, but the Nomenclature Committee on Cell Death (NCCD) categorised mitotic catastrophe as an oncosuppressive mechanism instead of a form of programmed cell death (Galluzzi et al., 2018). Indeed, others consider it as a process that leads to apoptosis or necrosis (Vakifahmetoglu et al., 2008). The current study showed that PDc can induce both mitotic catastrophe and oxeiptosis with ROS production as the common thread. It could be hypothesised that ROS production imbalanced the mitochondria homeostasis that led to the concomitant induction of oxeiptosis and formation of premature CDK1/Cyclin B1 complex, responsible for cell cycle arrest and mitotic catastrophe. Therefore, these findings open up interesting new perspectives on the induction of programmed cell death and cell cycle regulation in the treatment of cancer.

This study identified a novel combination that couples PEITC and dasatinib for an improved treatment of HCC through the induction of ROS-dependent cell cycle arrest and oxeiptosis. Taken together, these results highlight the role that isothiocyanates may have in cancer treatment by improving the anticancer activity of clinically approved chemotherapeutic agents.

## Data Availability

The raw data supporting the conclusion of this article will be made available by the authors, without undue reservation.
