# Supplementary material for: Phenethyl isothiocyanate and dasatinib combination synergistically reduces hepatocellular carcinoma growth via cell cycle arrest and oxeiptosis
Source: Front Pharmacol. 2023 Oct 4;14:1264032. doi: 10.3389/fphar.2023.1264032 (PMC10583560; doi:10.3389/fphar.2023.1264032)
Supplement: Supplementary file 1 [file DataSheet1.docx]

Supplementary Material

Phenethyl isothiocyanate and dasatinib combination synergistically reduces hepatocellular carcinoma growth via cell cycle arrest and oxeiptosis

Gabriele Strusi^1^ , Caterina M. Suelzu^1^ , Nicole Horwood^1^, Andrea E. Münsterberg^2^, *Yongping Bao^1^ .

^1^Norwich Medical School, University of East Anglia, Norwich Research Park, Norwich, UK

^2^School of Biological Sciences, University of East Anglia, Norwich, UK

*** Correspondence:**Yongping Bao
[y.bao@uea.ac.uk](mailto:y.bao@uea.ac.uk)

**Supplementary Figure 1.** Key resources

| **Product** | **Supplier** | **Identifier** |
| --- | --- | --- |
| **Materials** | | |
| CELLSTAR® cell repellent U bottom 96-well plates | Greiner Bio-One, Kremsmünster, Austria | 650970 |
| Round glass coverslips 15 mm | Scientific Laboratory Supplies, Nottingham, UK | MIC3338 |
| Square glass coverslips 18 mm | Chance Propper Ltd., West Midlands, UK |  |
| **Chemicals** | | |
| DMEM | Gibco, ThermoFisher Scientific, Loughborough, UK | 10938-025 |
| DMEM phenol red free | Gibco, ThermoFisher Scientific, Loughborough, UK | 21063-029 |
| MEM | Gibco, ThermoFisher Scientific, Loughborough, UK | 32561029 |
| FBS | Gibco, ThermoFisher Scientific, Loughborough, UK | 10500-064 |
| L-glutamine (200 mM) | Gibco, ThermoFisher Scientific, Loughborough, UK | 25030081 |
| Penicillin 100 U/ml Streptomycin (100 µg/ml) | Gibco, ThermoFisher Scientific, Loughborough, UK | 15070-063 |
| Trypsin EDTA 0.25%, 0.05% | Gibco, ThermoFisher Scientific, Loughborough, UK | 25200-056 |
| Goat serum | Merck Life Science, Gillingham, UK | G9023 |
| ECM Gel | Merck Life Science, Gillingham, UK | E6909 |
| PEITC | Merck Life Science, Gillingham, UK | 253731 |
| Dasatinib | Merck Life Science, Gillingham, UK | SML2589 |
| **Staining and histology** | | |
| Calcein AM | Merck Life Science, Gillingham, UK | C1359 |
| Ethidium homodimer-1 | Merck Life Science, Gillingham, UK | E1903 |
| ProLong™ Gold Antifade Mountant with DAPI | ThermoFisher Scientific, Loughborough, UK | P36931 |
| Rhodamine Phalloidin | ThermoFisher Scientific, Loughborough, UK | R415 |
| **Commercial assays** | | |
| DCFDA - Cellular ROS Assay Kit | abcam Inc., Cambridge, UK | ab113851 |
| BD Pharmingen™ PI/RNase staining | BD Biosciences, Franklin Lakes, New Jersey, USA | 550825 |
| Annexin V PI staining kit | Invitrogen, ThermoFisher Scientific, Loughborough, UK | V13242 |
| Apoptosis Human Proteome Profiler^TM^ | Bio-Techne Ltd, Abingdon, UK | ARY009 |
| **Western blotting** | | |
| Bradford reagent | Merck Life Science, Gillingham, UK | B6916 |
| Protease Inhibitor Cocktail (100X) | Cell Signaling Technologies, Leiden, NL | 5871S |
| Pierce™ Phosphatase Inhibitor Mini Tablets | ThermoFisher Scientific, Loughborough, UK | A32957 |
| 4x NuPage LDS sample buffer | Invitrogen, ThermoFisher Scientific, Loughborough, UK | NP0007 |
| TruPAGE™ TEA-Tricine SDS Running Buffer | Merck Life Science, Gillingham, UK | PCG30001 |
| PageRuler™ Plus | Invitrogen, ThermoFisher Scientific, Loughborough, UK | 26619 26616 |
| Immobilon FL PVDF | Merck Life Science, Gillingham, UK | IPFL00010 |
| TBS blocking buffer | LI-COR Biotechnology, Cambridge, UK | 927-60001 |
| IRDye® 800CW Streptavidin | LI-COR Biotechnology, Cambridge, UK | 926-32230 |
| Antibodies | Santa Cruz Biotech, Dallas, Texas, USA  R&D Systems, Bio-Techne Ltd, Abingdon, UK  Cell Signaling Technologies, Leiden, NL  ECM Biosciences, Aurora, Colorado, USA  Upstate, Merck Life Science, Gillingham, UK  LI-COR Biotechnology, Cambridge, UK | Supplementary Table 2  Supplementary Table 3 |
| **PCR** | | |
| Quick-DNA Microprep Kit | Zymo Research, Irvine, USA | D3021 |
| MassRuler™ DNA Ladder | ThermoFisher Scientific, Loughborough, UK | SM0403 |
| SYBR safe DNA gel stain | ThermoFisher Scientific, Loughborough, UK | S33102 |
| **Experimental models: cell lines and organisms** | | |
| HepG2 cell line | American Type Culture Collection (ATCC), Manassas, Virginia, USA | HB-8065 |
| Hepa 1-6 cell line | American Type Culture Collection (ATCC), Manassas, Virginia, USA | CRL-1830 |
| C57BL/6NHsd 6-7 Weeks | Envigo RMS, Bicester, UK | 11204F |
|  | | |
|  | | |
| **Critical Instruments** | | |
| EVOS M5000 Imaging System | ThermoFisher Scientific, Loughborough, UK | N/A |
| Leica DMI3000 B | Leica Biosystems Nussloch GmbH, Nußloch, Germany | N/A |
| IM IC5500 | Ricoh Company Ltd, Tokyo, Japan | N/A |
| FLUOstar Omega | BMG Labtech Ltd., Bucks, UK | N/A |
| BD FACSymphony A1™ | BD Biosciences, Franklin Lakes, New Jersey, USA | N/A |
| Cube 6 | Sysmex Partec GmbH, Goerlitz, Germany | N/A |
| GBOX Chemi XRQ | Syngene, Cambridge, UK | N/A |
| Odyssey CLx Imaging System | LI-COR Biotechnology, Cambridge, UK | N/A |
| Nanodrop 2000 | ThermoFisher Scientific, Loughborough, UK | N/A |
| Mini Protean Tank | Bio-Rad Laboratories, Hercules, California, USA | N/A |
| Trans-Blot® Turbo™ Transfer System | Bio-Rad Laboratories, Hercules, California, USA | N/A |
| Gilson HPLC-306 Laboratory system | Gilson, Middleton, Wisconsin, USA | N/A |
| HiChrom ACE-AR C18 | Phenomenex, Torrance, California, USA | N/A |
| Jasco FP-920 | Oklahoma City, Oklahoma, USA | N/A |
| **Software and algorithms** | | |
| GraphPad Prism 9 | GraphPad software, San Diego, California USA | v9.1.1 |
| Compusyn | ComboSyn Inc., Paramus, New Jersey USA | N/A |
| ModFit LT | Verity Software House, Topsham, Maine, USA | v6.0.11 |
| FlowJo 10.4 | BD Biosciences, Franklin Lakes, New Jersey, USA | v10.4 |
| Image Studio Lite 5.2.5 | LI-COR Biotechnology, Cambridge, UK | v5.2.5 |
| Clarity 2.6 | DataApex, Prague, Czech Republic | v2.6 |
| ImageJ 1.53k | National Institute of Health, Bethesda, Maryland, USA | v1.53k |

**Supplementary Table 2.** List of antibodies used in Western blot

|  | **Antigen** | **Host** | **Dilution** | **Molecular weight** | **Supplier** | **Catalogue number** |
| --- | --- | --- | --- | --- | --- | --- |
| **Primary antibodies** | Chk2 | Rabbit | 1:2000  (0.5 µg/mL) | 65 kDa | R&D Systems | MAB1358 |
|  | p-Chk2 (Thr68) | Rabbit | 1:400  (0.5 µg/mL) | 65 kDa | R&D Systems | AF1626 |
|  | p53 | Mouse | 1:1000  (0.2 µg/mL) | 53 kDa | Santa Cruz Biotech | sc-126 |
|  | p-p53 (S15) | Rabbit | 1:666  (0.13 µg/mL) | 53 kDa | R&D Systems | AF1043 |
|  | CDK1 | Mouse | 1:1000  (0.2 µg/mL) | 34 kDa | Santa Cruz Biotech | sc-54 |
|  | p-CDK1 | Rabbit | 1:666  (0.09 µg/mL) | 34 kDa | Cell Signaling Technologies | #9114 |
|  | Cyclin B1 | Mouse | 1:1000  (0.2 µg/mL) | 60 kDa | Santa Cruz Biotech | sc-245 |
|  | Caspase-9 | Rabbit | 1:1000  (0.2 µg/mL) | 46 kDa | Santa Cruz Biotech | sc-8355 |
|  | Caspase-3 | Rabbit | 1:1000  (0.2 µg/mL) | 32 kDa | Santa Cruz Biotech | sc-7148 |
|  | Cytochrome c | Goat | 1:2000  (0.1 µg/mL) | 15 kDa | Santa Cruz Biotech | sc-8385 |
|  | Keap1 | Goat | 1:1000  (0.2 µg/mL) | 69 kDa | Santa Cruz Biotech | sc-15246 |
|  | PGAM5 | Mouse | 1:1000  (0.2 µg/mL) | 32 kDa | Santa Cruz Biotech | sc-515880 |
|  | AIF | Rabbit | 1:6500  (0.1 µg/mL) | 66 kDa | R&D Systems | AF1457 |
|  | p-AIF (Ser116) | Rabbit | 1:1000  (0.2 µg/mL) | 66-57 kDa | ECM Biosciences | AP5501 |
|  | β-Actin | Mouse | 1:2000  (0.1 µg/mL) | 43 kDa | Santa Cruz Biotech | sc-47778 |
|  | β-Actin | Rabbit | 1:5000 | 43 kDa | Cell Signaling Technologies | #4970 |
| **Secondary antibodies** | IRDye^®^ 800CW Mouse | Donkey | 1:10000  (1 µg/mL) | N/A | LI-COR | 926-32212 |
|  | IRDye^®^ 800CW Rabbit | Donkey | 1:10000  (1 µg/mL) | N/A | LI-COR | 926-32213 |
|  | IRDye^®^ 800CW Goat | Donkey | 1:10000  (1 µg/mL) | N/A | LI-COR | 926-32214 |
| **Secondary antibodies** | IRDye^®^ 680LT Rabbit | Donkey | 1:10000  (1 µg/mL) | N/A | LI-COR | 926-68023 |
|  | IRDye^®^ 680LT Mouse | Goat | 1:10000  (1 µg/mL) | N/A | LI-COR | 926-68020 |

**Supplementary Table 3.** List of antibodies used in immunocytochemistry

|  | **Antigen** | **Dilution** | **Supplier** | **Catalogue number** |
| --- | --- | --- | --- | --- |
| **Primary antibodies** | ⍺-Tubulin | 1:200 | Santa Cruz Biotech | sc-5286 |
| **Secondary antibodies** | AlexaFluor 488 | 1:500 | Thermo Fisher Scientific | A-21202 |

**
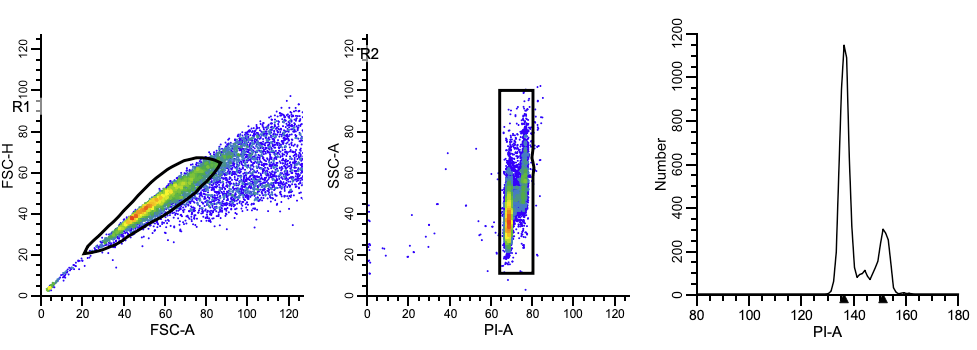
**

**Supplementary Figure 1.** Gating strategy for Propidium Iodide staining for detection of cell cycle phases in HepG2.

**
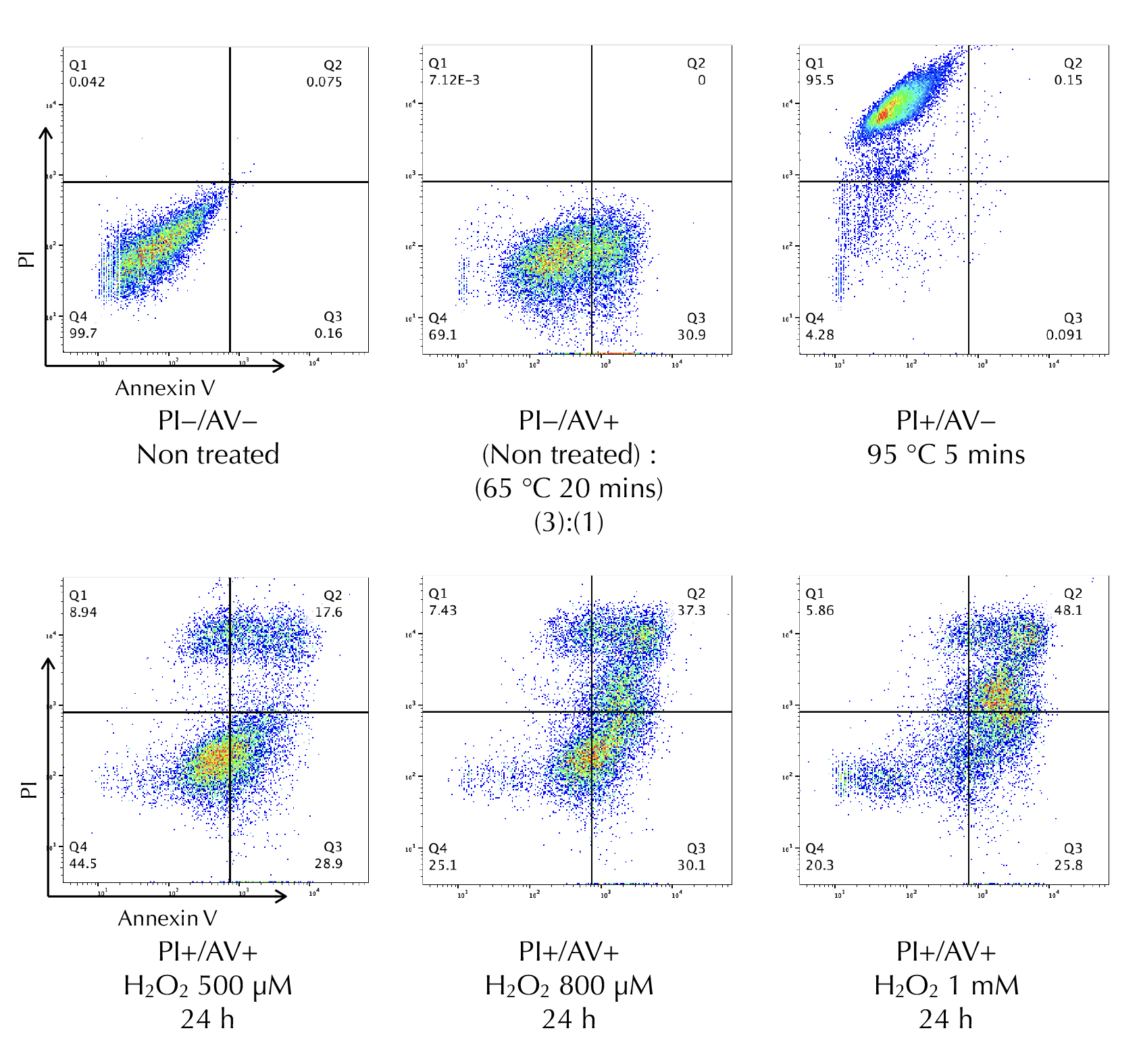
**

**Supplementary Figure 2.** Representative compensation data and positive control for Annexin V and Propidium Iodide apoptosis assay in HepG2.

**
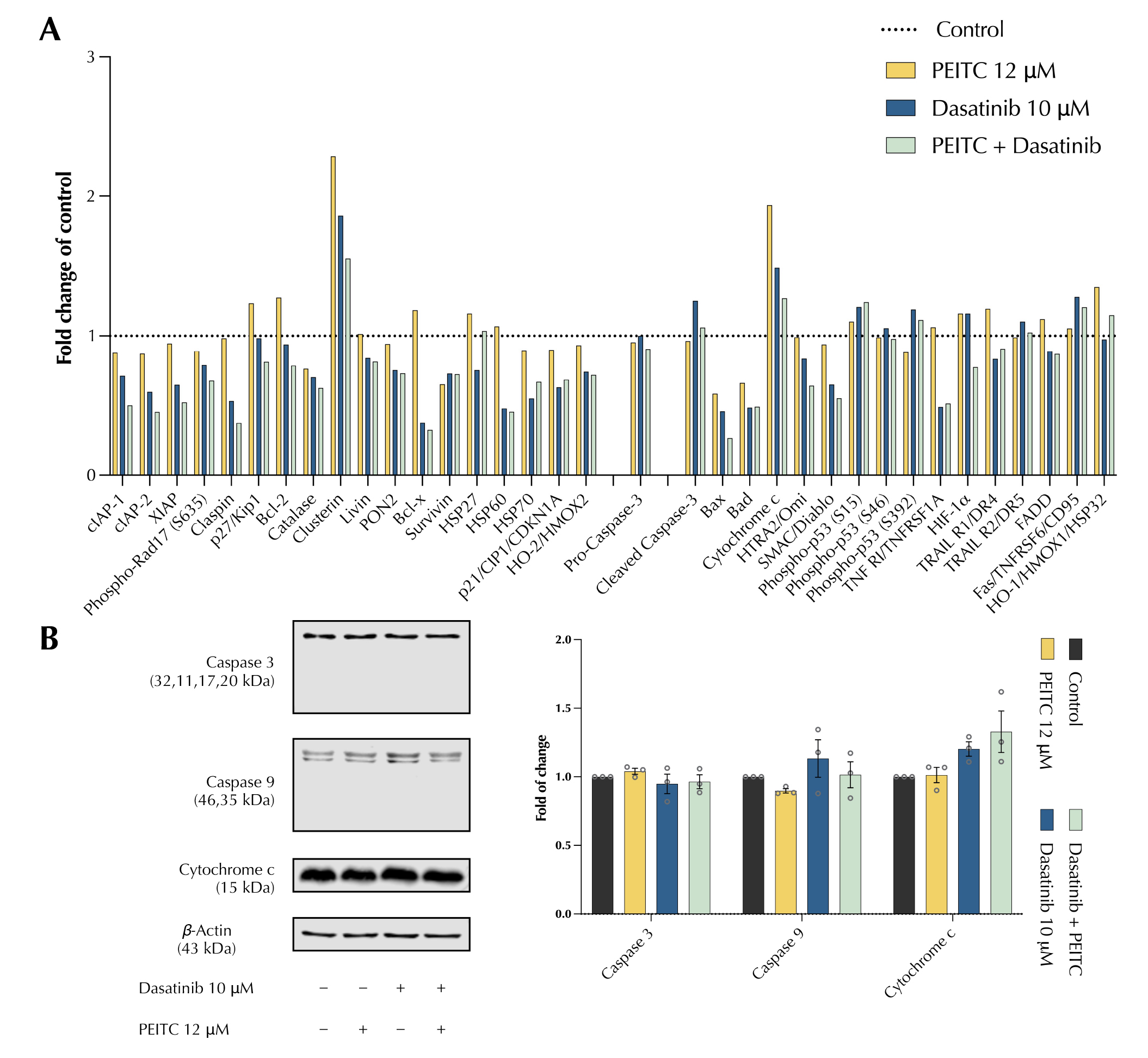
**

**Supplementary Figure 3.** PDc does not change expression of apoptosis markers in HepG2 HCC cells. (**A)** Proteome profiler for apoptosis markers; data presented in terms of fold change of control. **(B)** Western blot analysis of Caspase-3, Caspase-9, Cytochrome C; the results presented are representative of three independent protein extractions (left). Fold change to control normalised to β-Actin (mean ± SEM, n≥3) (right).

**Apoptosis Proteome Profiler**

In order to identify the factors involved in the PDc anti-apoptotic action, a Bio-Techne Proteome Profiler^TM^ Human Apoptosis Antibody Array was employed. As per manufacturer instructions 300 µg cell lysates were diluted and mixed with blocking buffer to a final volume of 1.5 mL. The membrane were incubated with the sample solution over night at 4 °C on a rocking platform. After that the membranes were washed three times 10 minutes each and incubated with a detection antibody cocktail for 1 h at room temperature on a rocking platform. Then the membranes were washed three times 10 minutes each and incubated with IRDye® 800CW Streptavidin for 30 minutes at room temperature. After 3 further washes of 10 minutes each the membranes were imaged with Odyssey CLx Imaging System. The dot density was quantified using Image Studio Lite 5.2.5 software and compared to the signal of a control membrane.


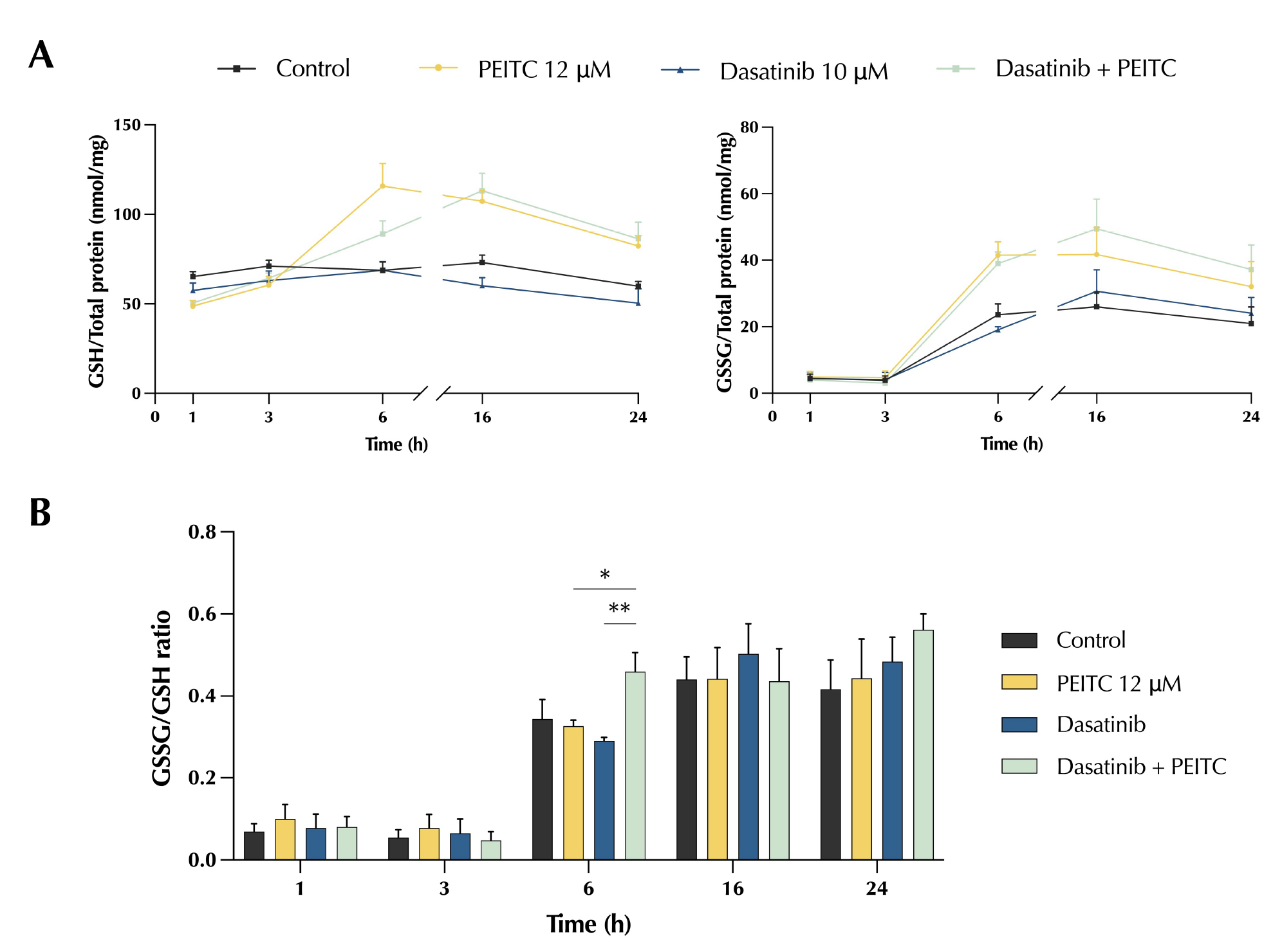


**Supplementary Figure 3.** Effect of PDc on GSSG/GSH ratio analysed with HLPC. (**A)** Quantification of GSH and GSSG over time. **(B)** GSSG/GSH ratio.

**GSSG/GSH levels quantification - HPLC assay**

HepG2 (1.8x10^5^ cells/well) were seeded in 6-well plates and once reached confluence different test compounds was added. At specified time points, cells were detached and resuspended in 75 µL of 5 mM diethylenetriaminepentaacetic acid (DTPA) in PBS. Samples were added with of 300 µl 50 mM methanesulfonic acid (MsOH), and then subjected to three freeze-thaw cycles alternating. GSSG/GSH-containing supernatants were obtained after centrifugation at 12,000 x g for 10 min. GSH was derivatized with monobromobimane mixing 75 µl of sample with 25 µl of reaction buffer composed of 50 mM HEPES pH 8, 5 mM EDTA, 15 mM NaOH, 2 mM mBBr, in acetonitrile. The reaction eas incubated for 15 min in the dark. The reaction was stopped through acidification with 1 µl 5 M MsOH and samples were stored at -20 °C after three-fold dilution with 10 mM MsOH. For GSSG isolation 150 µl of sample was mixed with 50 µl reaction buffer composed of 50 mM HEPES pH 8, 5 mM EDTA, 15 mM NaOH, 2 mM NEM, in acetonitrile. The reaction was incubated for 15 min in the dark. Disulfides were reduced for 20 min with 3 mM DTT. Reduced thiols from disulfides were derivatised in 7 mM mBBr for 15 min in the dark. The reaction was halted through acidification with 2 µl 5 M MsOH and samples were stored at -20 °C. The GSH/GSSG-mBBr complexes were separated by HPLC using a Gilson HPLC-306 Laboratory system with fluorescence detector Jasco FP-920 using the software Clarity 2.6. A HiChrom ACE-AR C18 4.6 × 250 mm, 5 µm column was equilibrated at 37°C with Solvent A (0.25% v/v acetic acid and 10% methanol, pH 4 NaOH). Samples were eluted with a gradient of Solvent B (90% methanol) at 1.0 ml/min flow rate as follow: 0-5 mins 0%, 5-15 mins 20%, 15-20 100% followed by 5 mins re-equilibration. The peak of GSH and reduced GSSG were observed at 7.9 min. Concentrations derived from interpolation from the standard curves were normalised to total protein concentration in initial sample considering the injection of 10 µl.


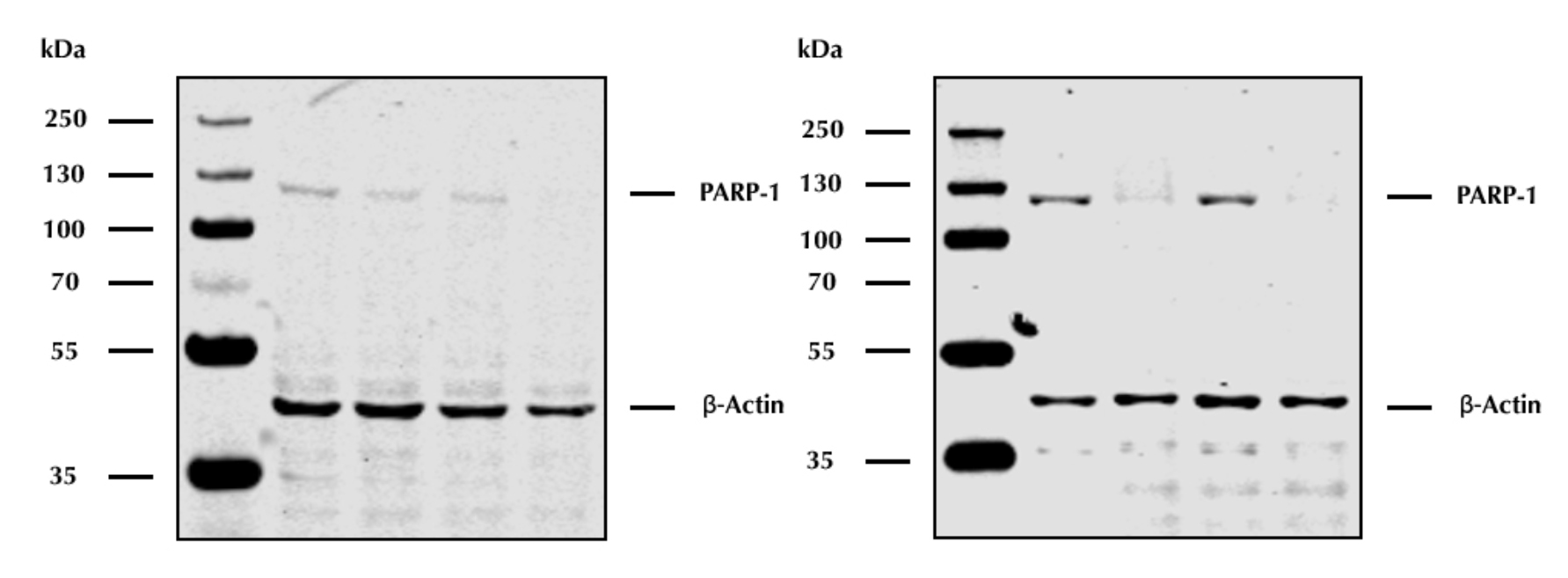


**Supplementary Figure 4.** Representative full blots of PARP1.

Samples order **Left**: Control, PEITC 12 µM, dasatinib 10 µM, PEITC + dasatinib. Samples order **right** Control, PEITC + dasatinib, NAC 20 mM, NAC + PEITC + dasatinib.


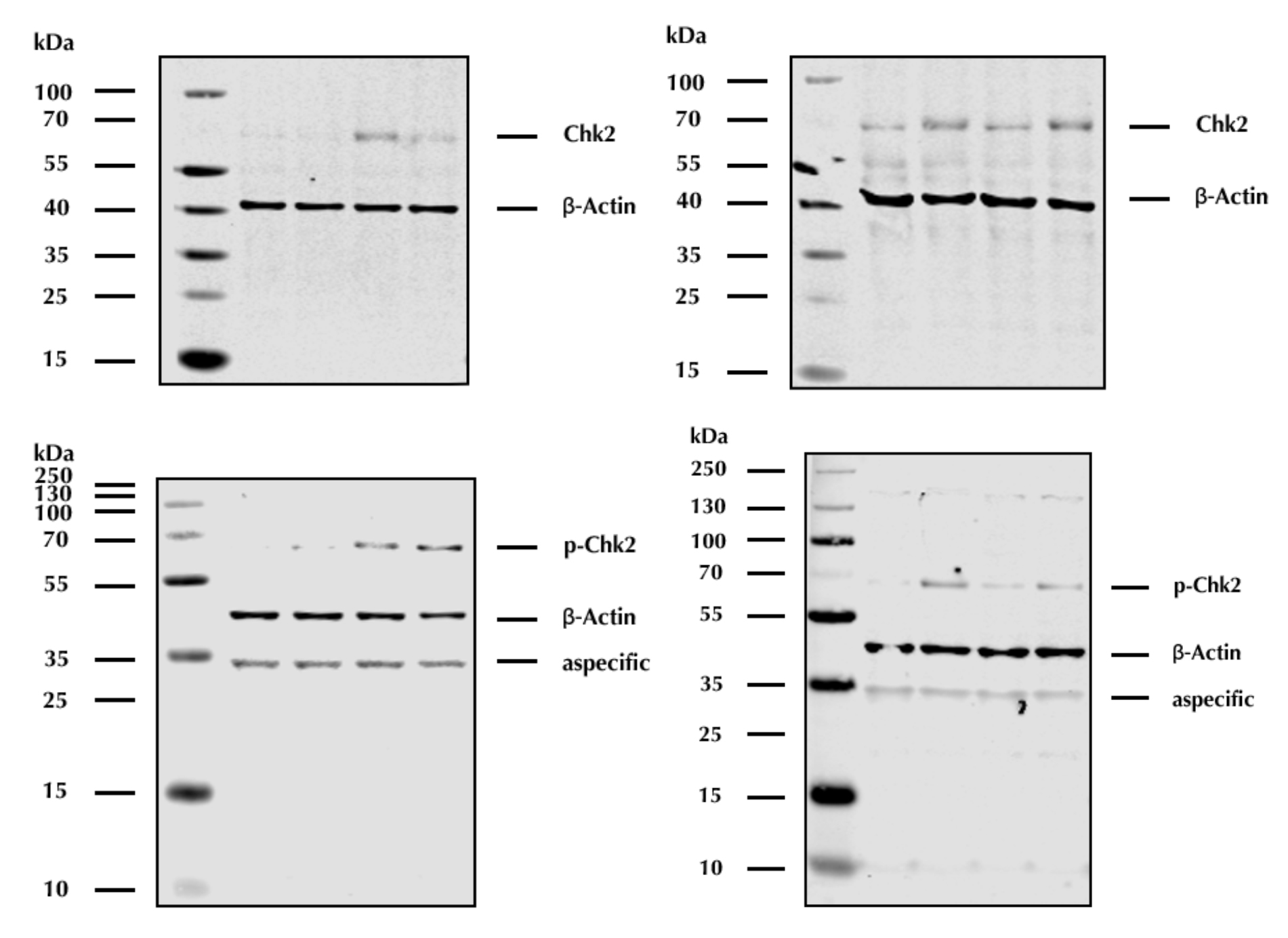


**Supplementary Figure 5.** Representative full blots of Chk2 and p-Chk2 (Thr-68).

Samples order **Left**: Control, PEITC 12 µM, dasatinib 10 µM, PEITC + dasatinib. Samples order **right:** Control, PEITC + dasatinib, NAC 20 mM, NAC + PEITC + dasatinib.


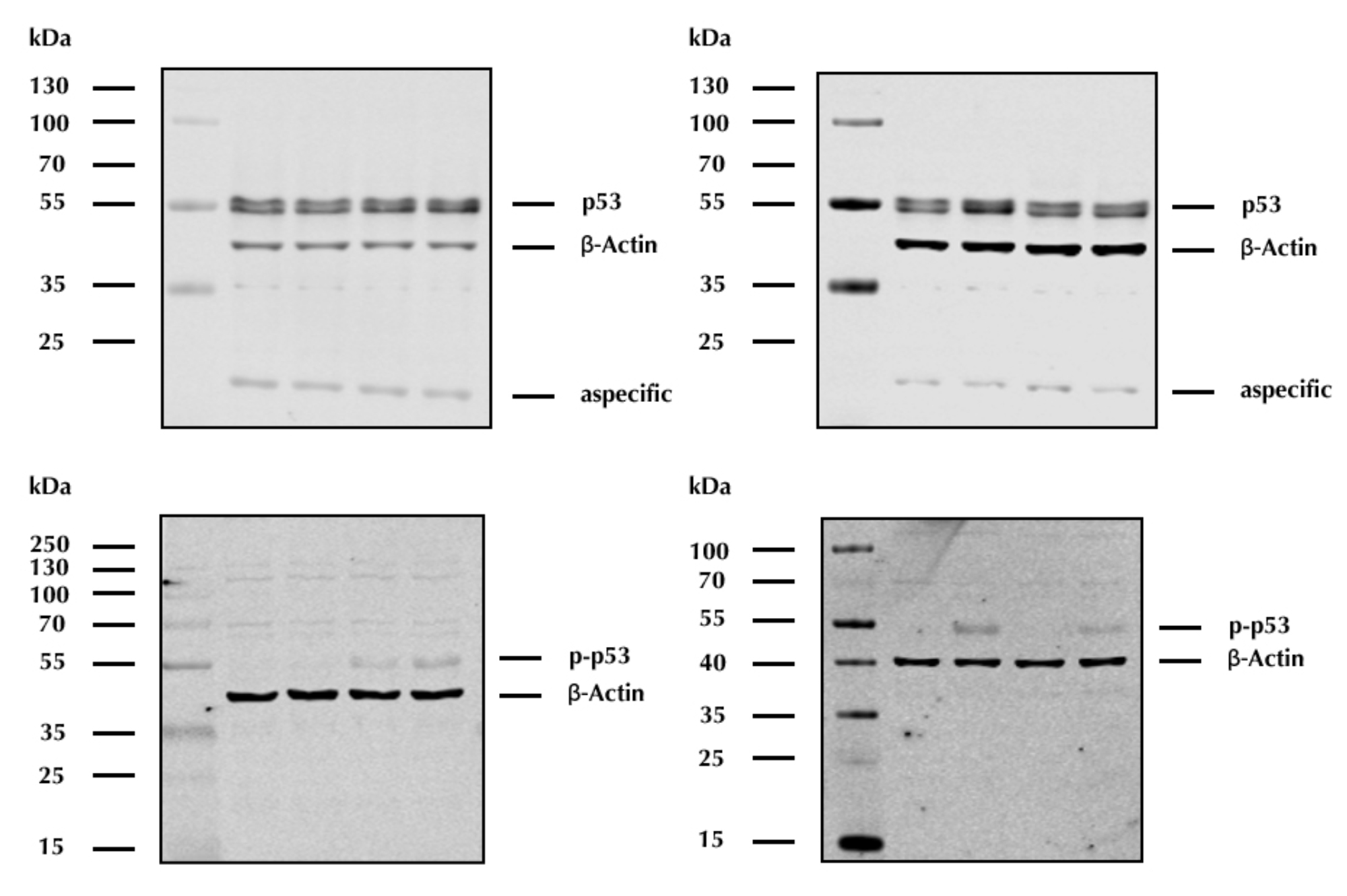


**Supplementary Figure 6.** Representative full blots of p53 and p-p53 (Ser-15)

Samples order **left**: Control, PEITC 12 µM, dasatinib 10 µM, PEITC + dasatinib. Samples order **right:** Control, PEITC + dasatinib, NAC 20 mM, NAC + PEITC + dasatinib.


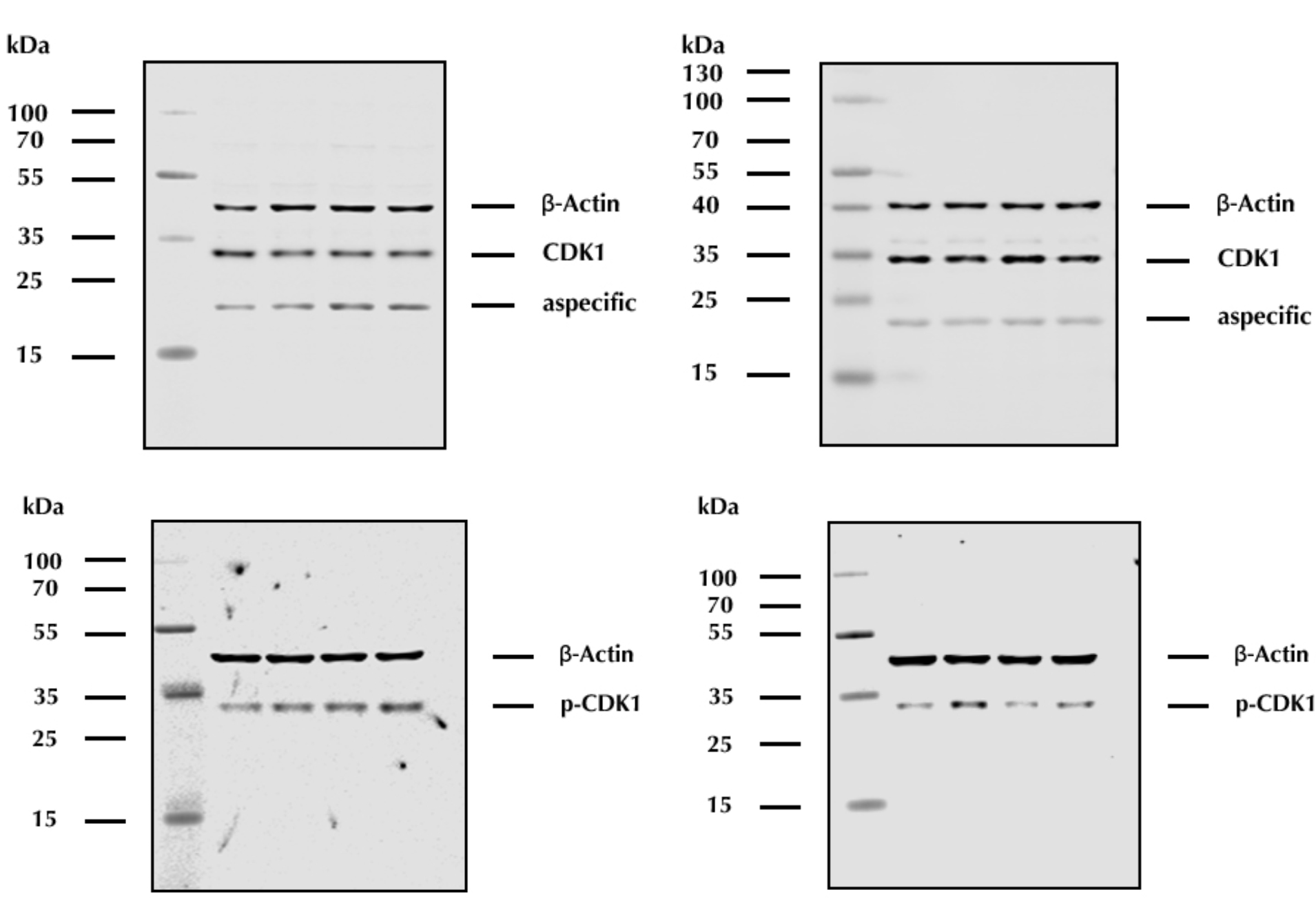


**Supplementary Figure 7.** Representative full blots of CDK1 and p-CDK1 (Thr-161).

Samples order **left**: Control, PEITC 12 µM, dasatinib 10 µM, PEITC + dasatinib. Samples order **right:** Control, PEITC + dasatinib, NAC 20 mM, NAC + PEITC + dasatinib.


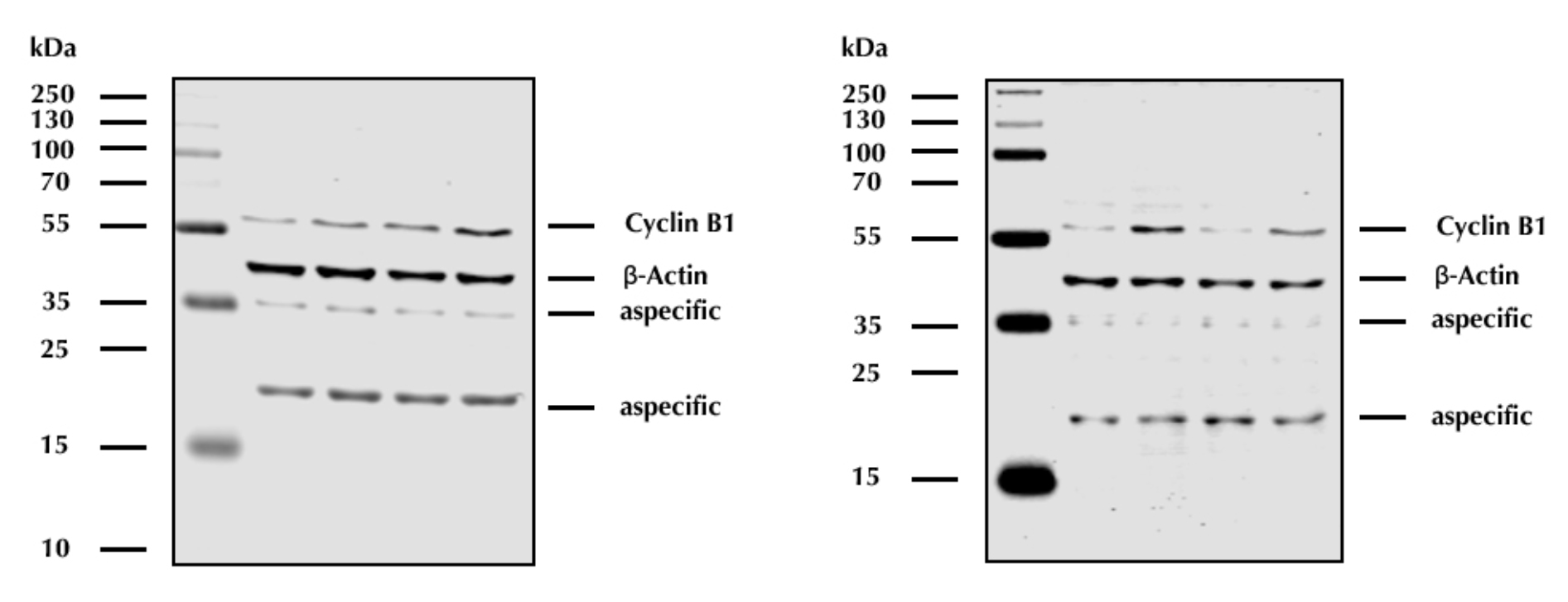


**Supplementary Figure 8.** Representative full blots of Cyclin B1.

Samples order **left**: Control, PEITC 12 µM, dasatinib 10 µM, PEITC + dasatinib. Samples order **right:** Control, PEITC + dasatinib, NAC 20 mM, NAC + PEITC + dasatinib.


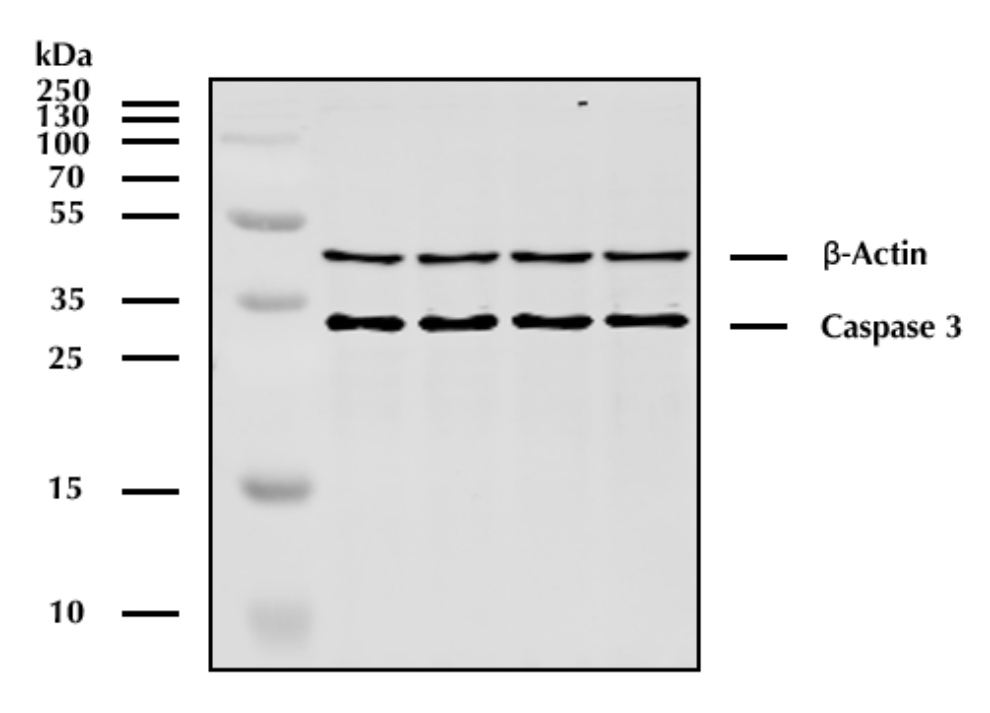


**Supplementary Figure 9.** Representative full blot of Caspase 3.

Samples order: Control, PEITC 12 µM, dasatinib 10 µM, PEITC + dasatinib.


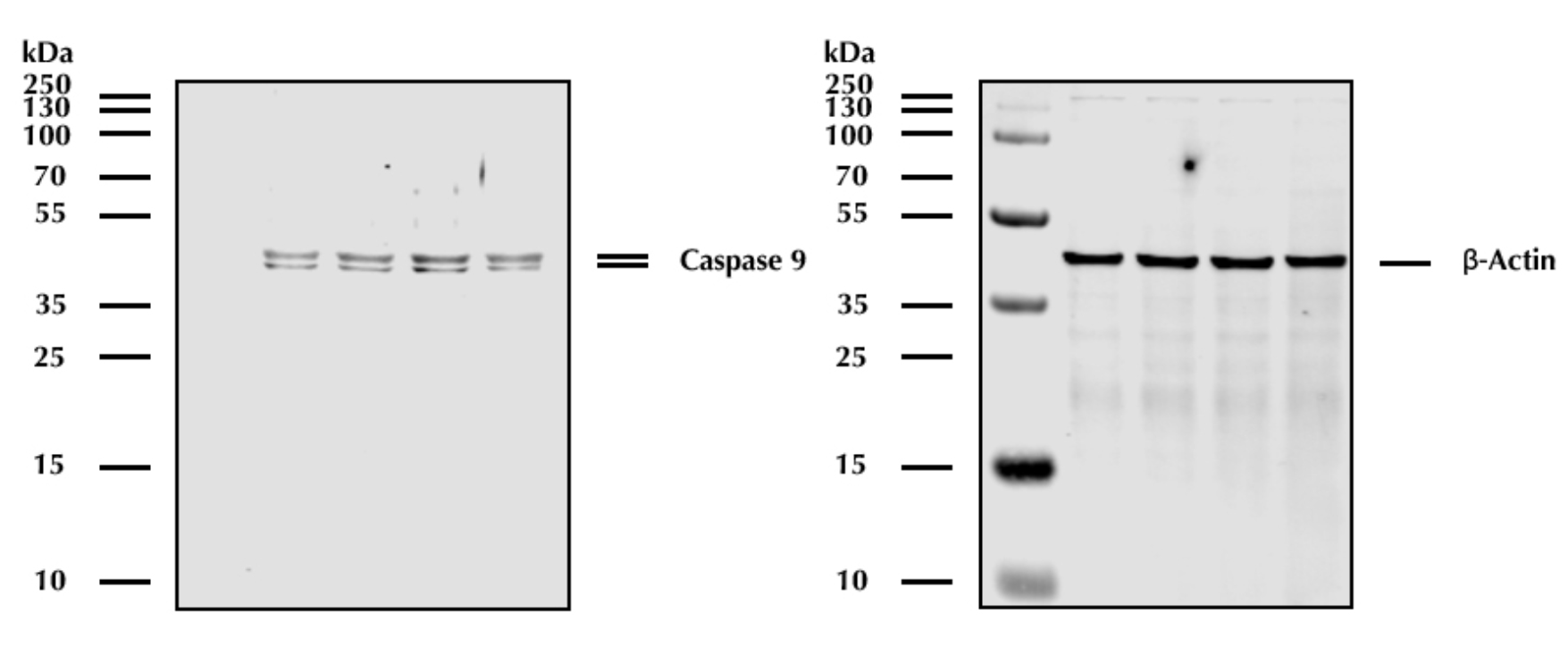


**Supplementary Figure 10.** Representative full blots of Caspase 9.

Samples order: Control, PEITC 12 µM, dasatinib 10 µM, PEITC + dasatinib. Caspase-9 and β-actin bands are on the same blot on different near infrared channels, but as they have similar molecular weight they cannot be on the same black and white image.


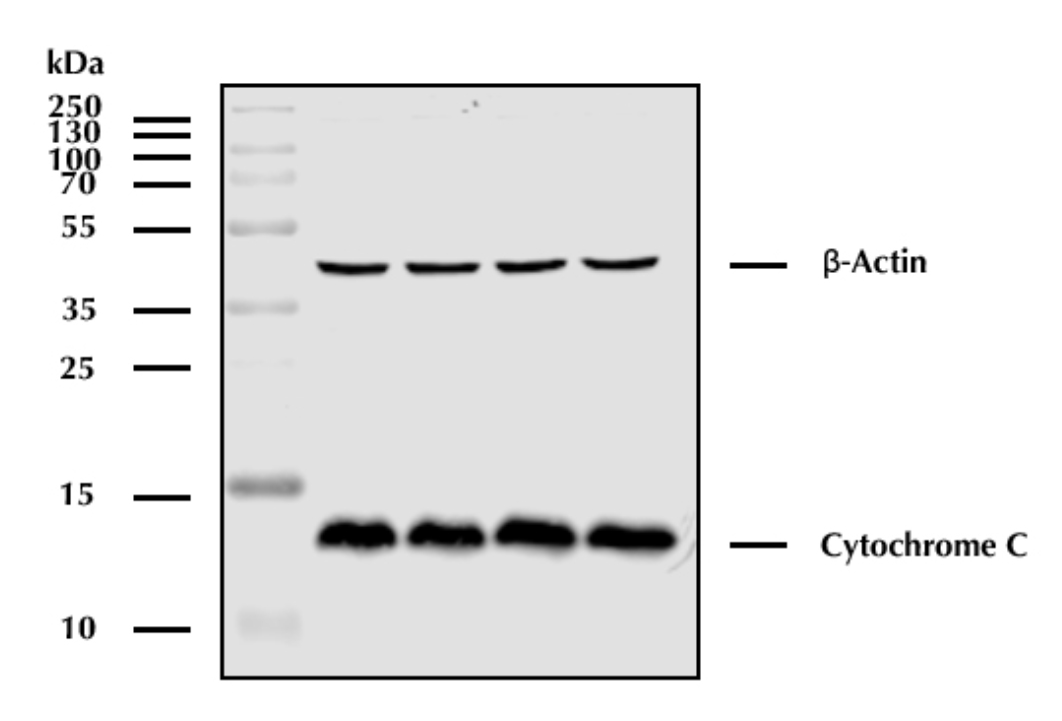


**Supplementary Figure 11.** Representative full blot of Cytochrome C.

Samples order: Control, PEITC 12 µM, dasatinib 10 µM, PEITC + dasatinib.


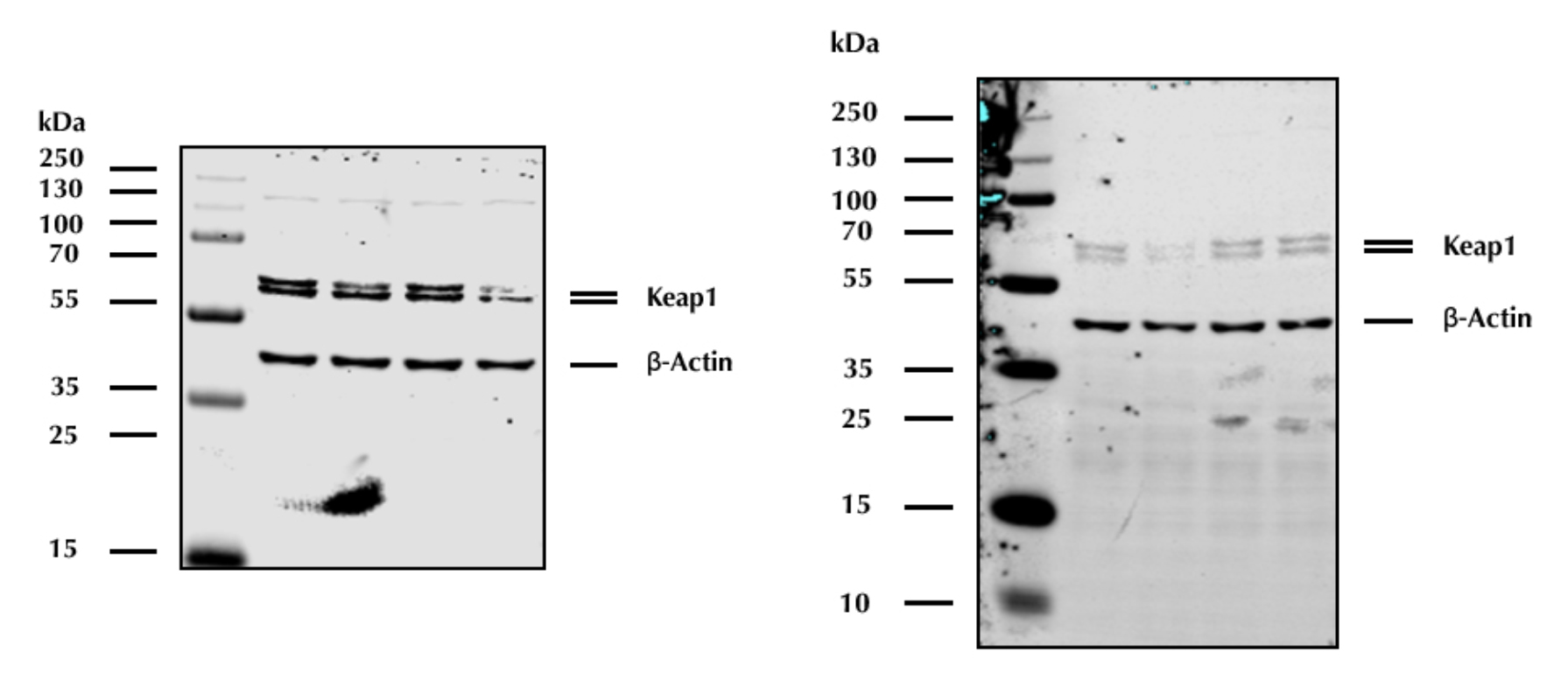


**Supplementary Figure 12.** Representative full blots of Keap1.

Samples order **left**: Control, PEITC 12 µM, dasatinib 10 µM, PEITC + dasatinib. Samples order **right:** Control, PEITC + dasatinib, NAC 20 mM, NAC + PEITC + dasatinib.


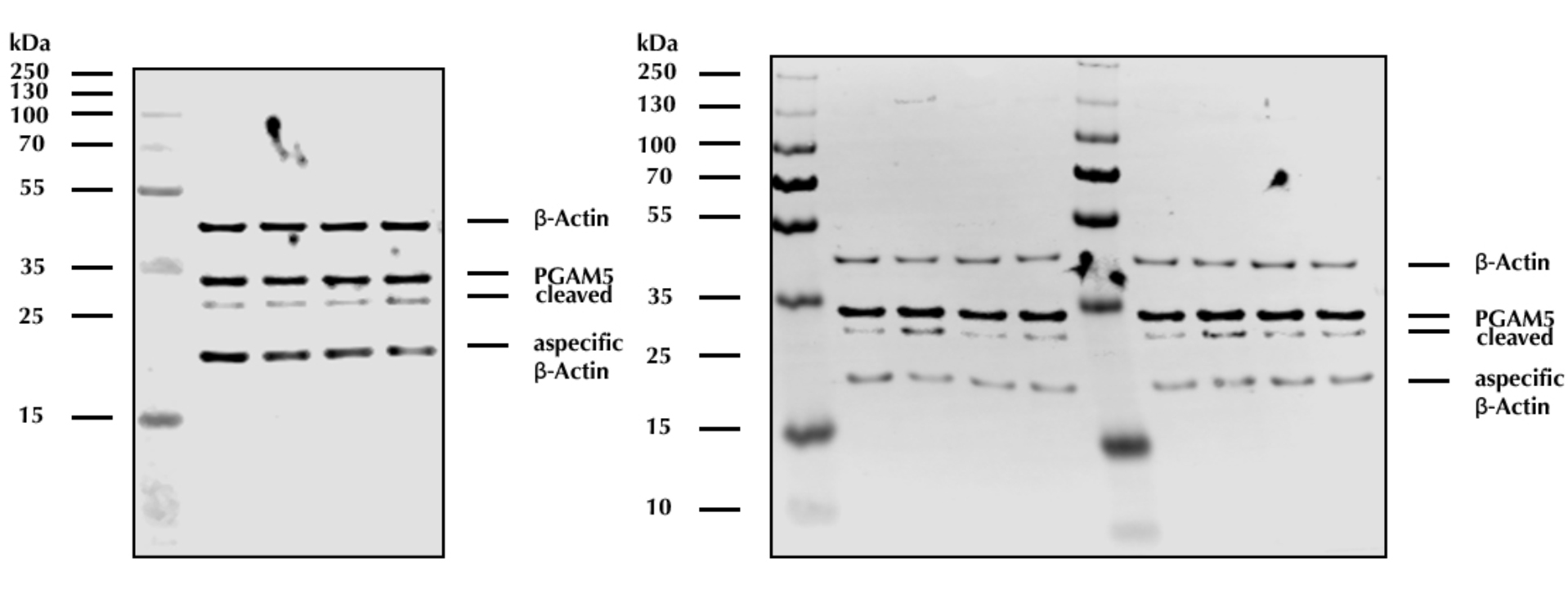


**Supplementary Figure 13.** Representative full blots of PGAM5.

Samples order **left**: Control, PEITC 12 µM, dasatinib 10 µM, PEITC + dasatinib. Samples order **right:** Control, PEITC + dasatinib, NAC 20 mM, NAC + PEITC + dasatinib.


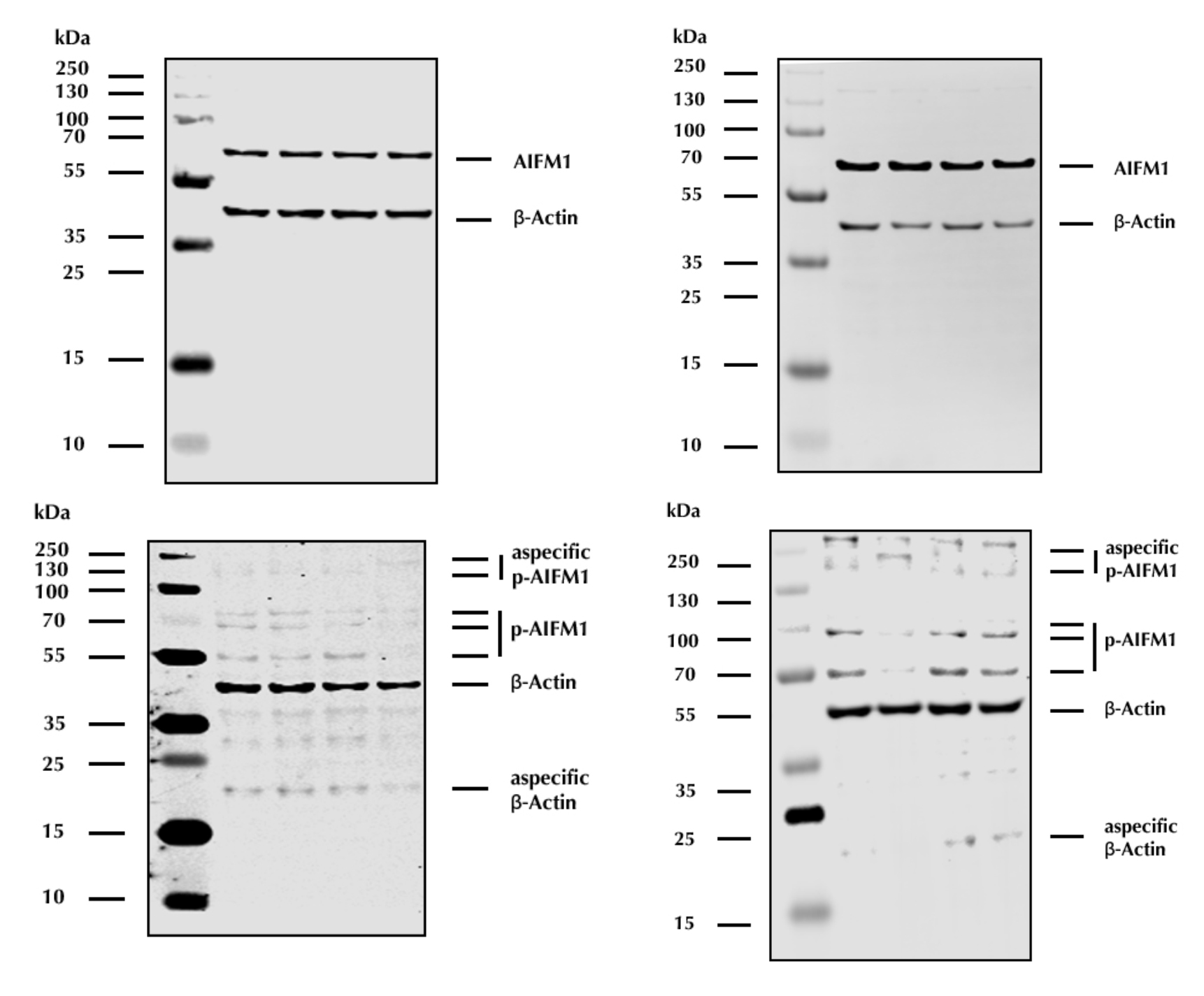


**Supplementary Figure 14.** Representative full blots of AIFM1 and p-AIFM1 (Ser-116). Samples order **left**: Control, PEITC 12 µM, dasatinib 10 µM, PEITC + dasatinib. Samples order **right:** Control, PEITC + dasatinib, NAC 20 mM, NAC + PEITC + dasatinib.

**
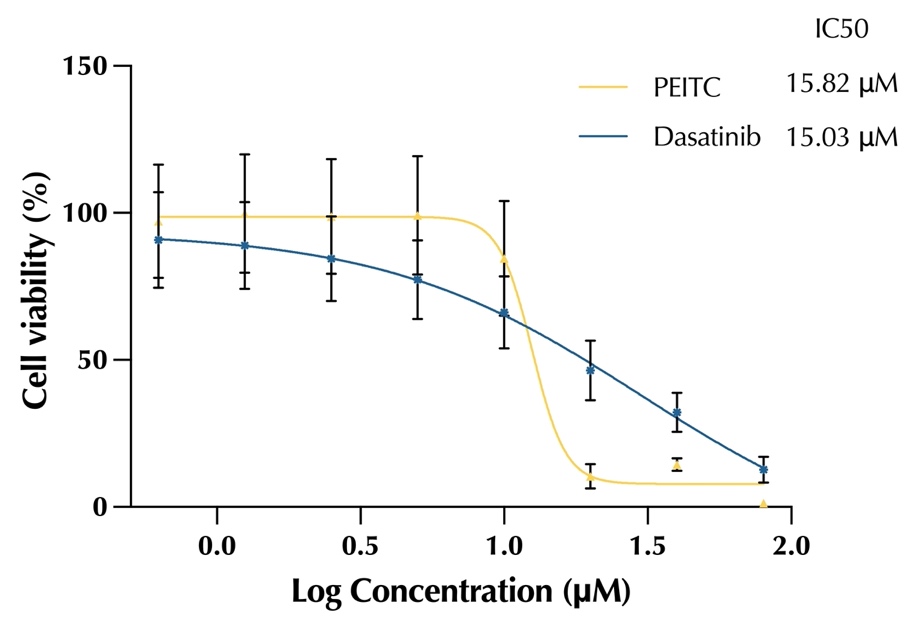
**

**Supplementary Figure 15.** Dose-response and IC50 values at 24 h for PEITC and dasatinib in HepG2 cells. IC50 values were calculated with CompuSyn software.

**
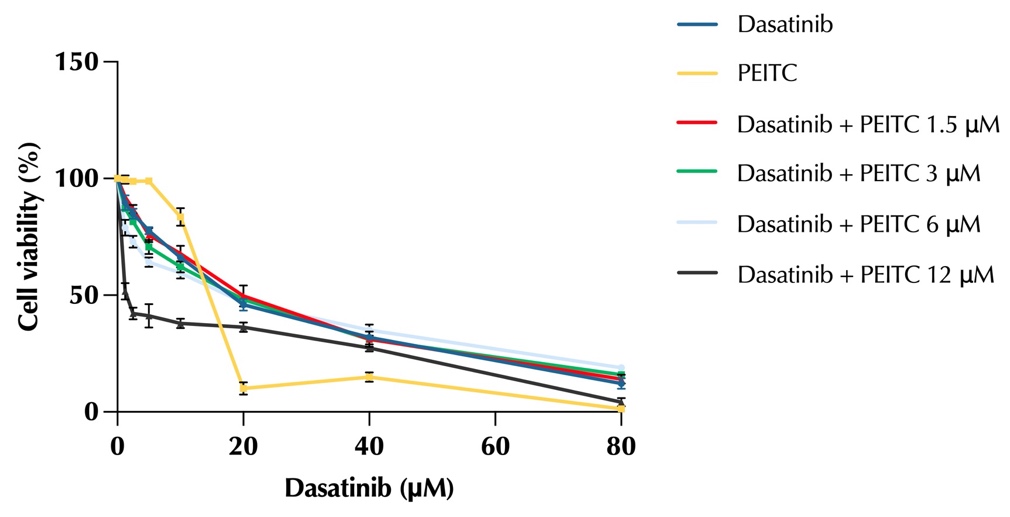
**

**Supplementary Figure 16.** Dose-response for lower concentrations of PEITC and dasatinib in HepG2 cells at 24 h.

**
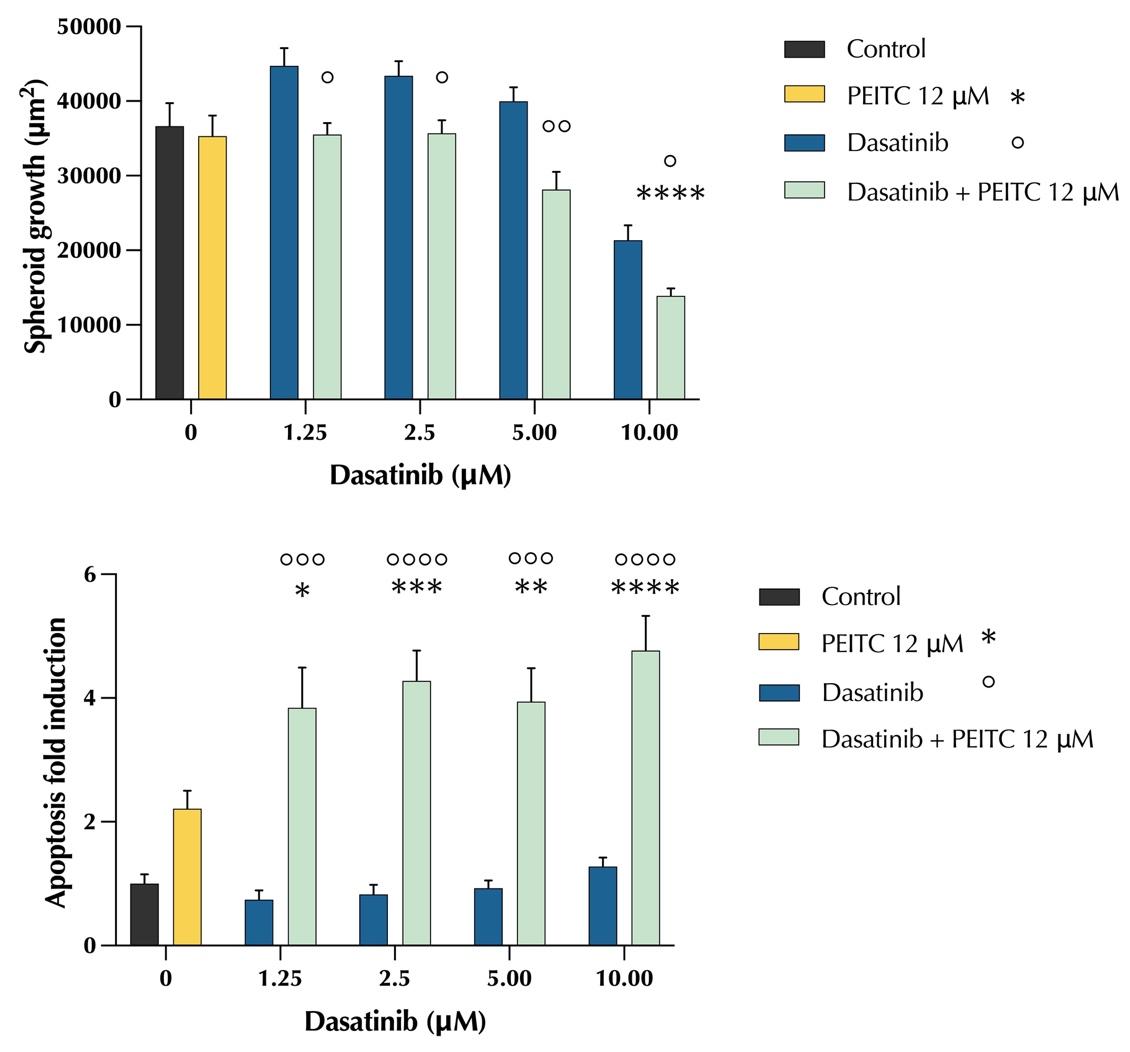
**

**Supplementary Figure 17.** Effect of lower concentrations of dasatinib on 3D spheroids growth and induction of apoptosis. Dasatinib 10 µM showed the most effective inhibition of 3D spheroids growth and induction of apoptosis when combined with PEITC; thus, this concentration was selected for mechanistic stud.

**Immunostaining of F-actin.**

HepG2 cells were seeded on 15 mm coverslips in 24 well plates (5x10^4^ cells/well). After 24 h cells were treated for further 24 h, fixed with 10% formalin for 15 mins and permeabilised with 0.1% Triton X-100. Blocking buffer (5% goat serum PBS) was added for 2 h on a rocker at room temperature. Phalloidin staining was added for 15 minutes at room temperature. The wells were rinsed in washing buffer and then washed twice for 5 minutes on a rocker at room temperature. Coverslips were mounted with 10 µL of ProLong™ Gold Antifade Mountant with DAPI. Microtubule morphology was observed using a Leica DMI3000 B microscope. Details of materials used are provided in Supplementary Table 1.

**
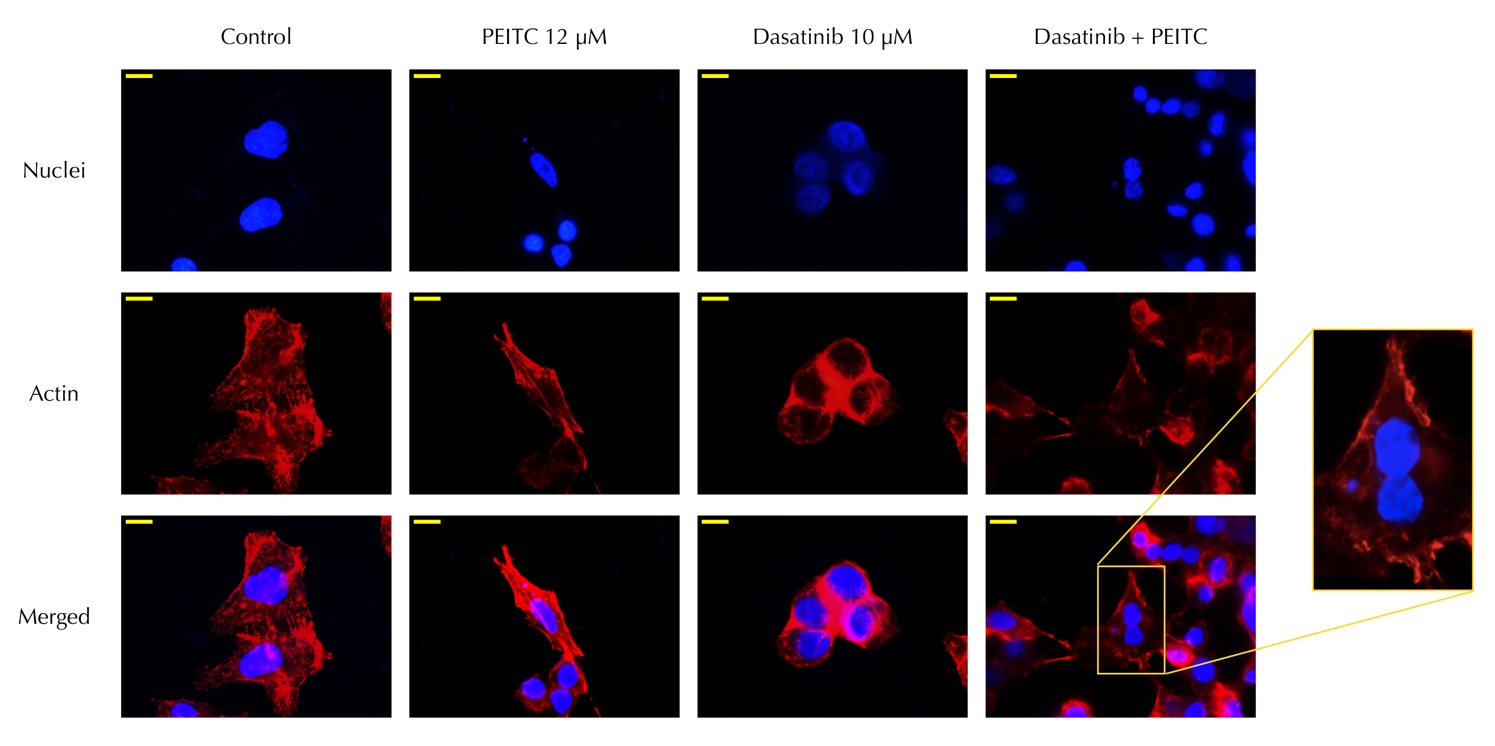
**

**Supplementary Figure 18.** Phalloidin staining of F-actin that underline the presence of multinucleated cells after treatment with PDc.

**DNA fragmentation assay**

Cells (HepG2 1.8x10^5^ cells/well) were seeded in 6-well plates and, once confluent, treatments were applied. After 24 h, DNA was extracted with Zymo Research Quick-DNA Microprep Kit and quantified. 2 ng of DNA were loaded into a 1.2% agarose gel added with 4 µL of SYBR safe DNA gel stain and run at 120 V for 120 min. DNA fragmentation was observed with a GBOX Chemi XRQ transilluminator.


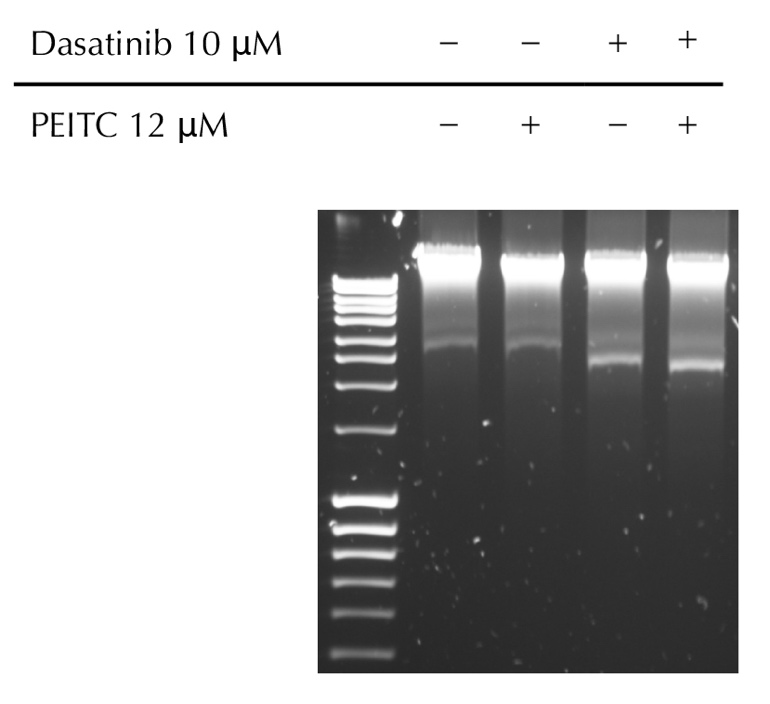


**Supplementary Figure 19.** DNA laddering assay shows partially laddering that indicate DNA fragmentation. The smear of combination treatment appears brighter and to go further in the gel run as its degradation results more marked.
